# Structural and Biophysical Profiling of HLA Class II Mismatches Using AlphaFold 3: A Computational Study

**DOI:** 10.3390/ijms27219442

**Published:** 2026-10-23

**Authors:** Vladyslav Bardash, Oleh Samchuk, Yaroslav Tolstyak, Hanna Demydenko, Eugen Sklyarov

**Affiliations:** 1 St. Panteleimon Hospital, First Lviv Medical Union, 79059 Lviv, Ukraine; 2 Department of Clinical Immunology and Allergology, Danylo Halytsky Lviv National Medical University, 79010 Lviv, Ukraine; 3 Department of Therapy No. 1, Medical Diagnostics, Hematology, and Transfusiology, Faculty of Postgraduate Education, Danylo Halytsky Lviv National Medical University, 79010 Lviv, Ukraine

**Keywords:** HLA, histocompatibility, AlphaFold 3, eplets, kidney transplantation, structural bioinformatics, precision medicine

## Abstract

Conventional linear eplet-matching algorithms (HLAMatchmaker, HLAMM) evaluate primary sequence polymorphisms across human leukocyte antigen (HLA) loci without integrating three-dimensional (3D) steric accessibility and local physicochemical constraints. Here, we developed an exploratory computational antigenicity pipeline using AlphaFold 3 to evaluate 10,000 simulated European donor–recipient pairs across pre-computed, full-length HLA class I (HLA-A, -B, -C) and heterodimeric class II (HLA-DR, -DQ, -DP) complexes modeled in a standardized peptide-free conformation. Candidate eplets were sequentially screened using a spatial solvent-accessible surface area threshold (SASA ≥ 10.0 Å^2^) and an empirical biophysical exclusion gate (5th percentile of absolute net charge |*Q*_net_| ≥ 0.3326 e and hydropathy *H*_total_ ≥ −7.265, calibrated on *N* = 170 antibody-verified eplets). Across the simulated cohort (*n* = 10,000), filtration reduced median [Q1; Q3] eplet counts from 19 [12; 26] to 17 [10; 23] for HLA-A, 22 [15; 29] to 18 [12; 24] for HLA-B, 12 [7; 17] to 10 [7; 15] for HLA-C, 15 [10; 19] to 11 [7; 14] for HLA-DR, 12 [6; 17] to 9 [4; 14] for HLA-DQ, and 7 [3; 12] to 6 [2; 9] for HLA-DP. Component ablation demonstrated that 3D steric accessibility (SASA ≥ 10.0 Å^2^) accounted for the vast majority of eplet exclusions—87.8% in HLA-DR and HLA-DP and 58.2% to 69.1% across HLA-DQ and class I loci—whereas the registry-calibrated biophysical gate acted as a secondary refinement. Within the subset of simulated pairs initially exceeding the arbitrary ≥10-eplet linear cutoff, the proportion dropping below 10 eplets after filtering was low for class I (HLA-A: 4.4%, HLA-B: 8.0%, HLA-C: 13.2%) but substantially higher for class II high-load subsets: HLA-DQ (21.8%), HLA-DR (26.0%), and HLA-DP (33.7% of 3693 baseline high-load pairs). Furthermore, class II loci, particularly HLA-DP, exhibited weakened covariance between heuristic charge and hydropathy indices that persisted after 1:1 count-matched subsampling (*ρ*_matched_ = −0.268) and partial correlation adjustment (*ρ*_partial_ = −0.385). This dual-filter pipeline defines a restricted mathematical subset of spatially accessible and biophysically suprathreshold candidate eplets. Because this is a hypothesis-generating in silico framework, multicenter retrospective registry analyses and prospective clinical trials evaluating de novo donor-specific antibody formation, biopsy-proven rejection, and allograft survival are strictly required before clinical translation.

## 1. Introduction

Alloimmune risk stratification in kidney transplantation has progressed from conventional serological antigen typing to refined assessments of molecular compatibility at the level of eplets—discrete polymorphic surface patches capable of eliciting antibody recognition [1,2]. Algorithms based on linear sequence alignment, most notably HLAMatchmaker (HLAMM), have become standard tools for estimating the likelihood of donor-specific antibody (DSA) development and forecasting graft outcomes [3,4]. Although complementary frameworks such as PIRCHE-II have enhanced predictions of indirect T-cell alloreactivity [5,6], accurately quantifying B-cell-relevant surface accessibility remains a persistent challenge.

Despite widespread adoption, these approaches fundamentally rely on a one-dimensional (1D) analysis of the human leukocyte antigen (HLA) primary amino-acid sequence. This simplification disregards the intrinsic three-dimensional (3D) structure of HLA molecules and, consequently, may misclassify buried, structurally constrained, or sterically inaccessible residues as immunogenic [7]. Such oversimplification can lead to inflated risk assessments and the potential exclusion of potentially compatible donors [8,9].

Recently, several in silico approaches have sought to incorporate 3D structural data to overcome these limitations. For example, the Snowflake algorithm utilizes neural networks to predict allele-specific solvent-accessible surface area (SASA), while toolkits like HLA3D provide comprehensive structural alignment databases [10,11]. Furthermore, dynamic models such as HLA-EpiCheck have employed molecular dynamics to extract 3D-surface patch descriptors [12]. However, these tools often rely on heterogeneous datasets of existing X-ray crystals, function primarily as visual databases without quantitative clinical risk reclassification or require immense computational resources that are currently impractical for high-throughput, real-time clinical cross-matching.

In this context, it is critical to operationally distinguish between antigenicity and immunogenicity [8,13]. For the purposes of this study, antigenicity is defined as the theoretical structural accessibility and physicochemical capacity of a polymorphic surface patch to bind an antibody. Conversely, immunogenicity encompasses the actual in vivo biological cascade culminating in a measurable clinical outcome, such as DSA formation or allograft rejection. Since static in silico models cannot simulate complete biological responses, our computational framework is strictly designed to evaluate theoretical antigenicity.

Amid the expanding application of artificial intelligence in transplantation medicine [14], the introduction of AlphaFold 3 has marked a significant advance in structural immunobiology. Utilizing a generative diffusion architecture, unlike previous generations, AlphaFold 3 achieves near-atomic accuracy in predicting biomolecular complexes [15] and shows markedly improved performance in reconstructing protein–protein interfaces fundamental to class II heterodimers (HLA-DR, -DQ, -DP) [15,16]. This capability enables a transition from theoretical enumeration of polymorphic substitutions to rigorous physicochemical characterization of molecular surfaces, incorporating electrostatic potential and hydrophobicity of epitopes in their native conformation, metrics previously established as relevant surrogate markers for HLA alloantigen immunogenicity [12,17,18].

In this study, we describe an automated, exploratory histocompatibility assessment pipeline grounded in full-length structural models generated by AlphaFold 3. Using a simulated European cohort of 10,000 donor–recipient pairs across all six classical HLA loci (class I: HLA-A, -B, -C; class II: HLA-DR, -DQ, -DP), with a primary analytical focus on heterodimeric class II molecules where conformational complexity is greatest, we developed a scalable, crystal-independent filtration algorithm. Extending beyond steric accessibility alone, our pipeline applies a sequential dual-filter framework: it first removes sterically occluded residues (SASA < 10.0 Å^2^) and subsequently applies an empirical biophysical exclusion gate based on electrostatic charge and hydropathy thresholds calibrated on antibody-verified eplets. Rather than constituting a validated clinical risk restaging, this dual filter isolates a restricted mathematical subset of candidate eplets that satisfy both spatial exposure and heuristic physicochemical criteria. Consequently, this hypothesis-generating computational framework is designed to prioritize candidate antigenic targets for subsequent empirical validation in multicenter retrospective registry analyses and prospective clinical trials evaluating de novo DSA development, biopsy-proven rejection, and allograft survival.

## 2. Results

### 2.1. Structural Validation and Model Quality

To ensure uniform structural fidelity for downstream physicochemical profiling, we generated full-length 3D structural models across all six classical HLA loci using the official web-based AlphaFold 3 Server (Google DeepMind) [15], comprising an 80-model structural repository (15 HLA-A, 15 HLA-B, 14 HLA-C, 11 HLA-DR, 10 HLA-DQ, and 15 HLA-DP complexes; Supplementary Table S6), of which 76 high-frequency European alleles and *α*/*β* haplotypes (population frequency ≥1.0%; Supplementary Table S13) were sampled in the 10,000-pair simulation. All class I heavy chain–*β*_2_ − microglobulin (*β*_2_m) complexes and class II *α*/*β* heterodimers were co-modeled in a standardized peptide-free conformation. Beyond preventing sequence-specific peptide bias across the simulated cohort, modeling empty binding clefts provides an informative, conservative upper-bound estimate of solvent-accessible surface area (SASA_empty_ ≥ SASA_peptide−loaded_) for groove-interior residues: any polymorphic residue on the *β*-sheet floor that is already sterically occluded by the overarching *α*-helical walls in the empty state (SASA < 10.0 Å^2^) would experience even greater steric shielding in the presence of a bound peptide in vivo.

#### 2.1.1. Internal Quality Assessment

First, we evaluated the intrinsic confidence metrics of the generated models across the entire structural library and stratified by individual locus (Supplementary Table S6). The library demonstrated consistently high prediction confidence across all loci, with an overall mean predicted Local Distance Difference Test (pLDDT) score of 86.50 ± 0.82 (per-locus mean pLDDT ranging from 85.65 ± 0.56 for HLA-A to 87.28 ± 0.57 [median 87.25, IQR 86.85–87.48] for HLA-B), while the folded extracellular antigen-presenting domains exhibited median residue-level pLDDT scores of 95.1–98.6 (contrasting with the lower-confidence, intrinsically flexible transmembrane and cytoplasmic tails). To assess inter-residue and inter-domain spatial accuracy derived from the AlphaFold 3 Predicted Aligned Error (PAE) matrices, we analyzed both the predicted Template Modeling (pTM) score and the interface pTM (ipTM) score (Supplementary Table S6). Because pTM and ipTM directly integrate the PAE matrix across intra-chain domains and inter-chain interfaces, the high overall median pTM of 0.81 [0.79; 0.81] (0.81 [0.81; 0.82] for class I and 0.78–0.79 for class II) and overall median ipTM of 0.94 [0.78; 0.94] (0.94 [0.94; 0.94] across class I heavy chain–β2m complexes and 0.78–0.80 across class II α/β heterodimers: HLA-DR 0.78 [0.78; 0.79], HLA-DQ 0.80 [0.79; 0.80], and HLA-DP 0.78 [0.77; 0.78]) confirm low positional uncertainty (< 1.5–3.0 Å PAE) throughout the folded extracellular core, whereas elevated PAE values are confined strictly to the unanchored C-terminal transmembrane linkers that do not participate in eplet formation. Global model confidence, represented by the ranking score, showed an overall median of 0.98 [0.88; 0.99] (0.99 [0.99; 0.99] for class I and 0.88–0.89 for class II), confirming high prediction confidence across all modeled conformations.

#### 2.1.2. Benchmarking Against Experimental Structures

**Figure 1:**
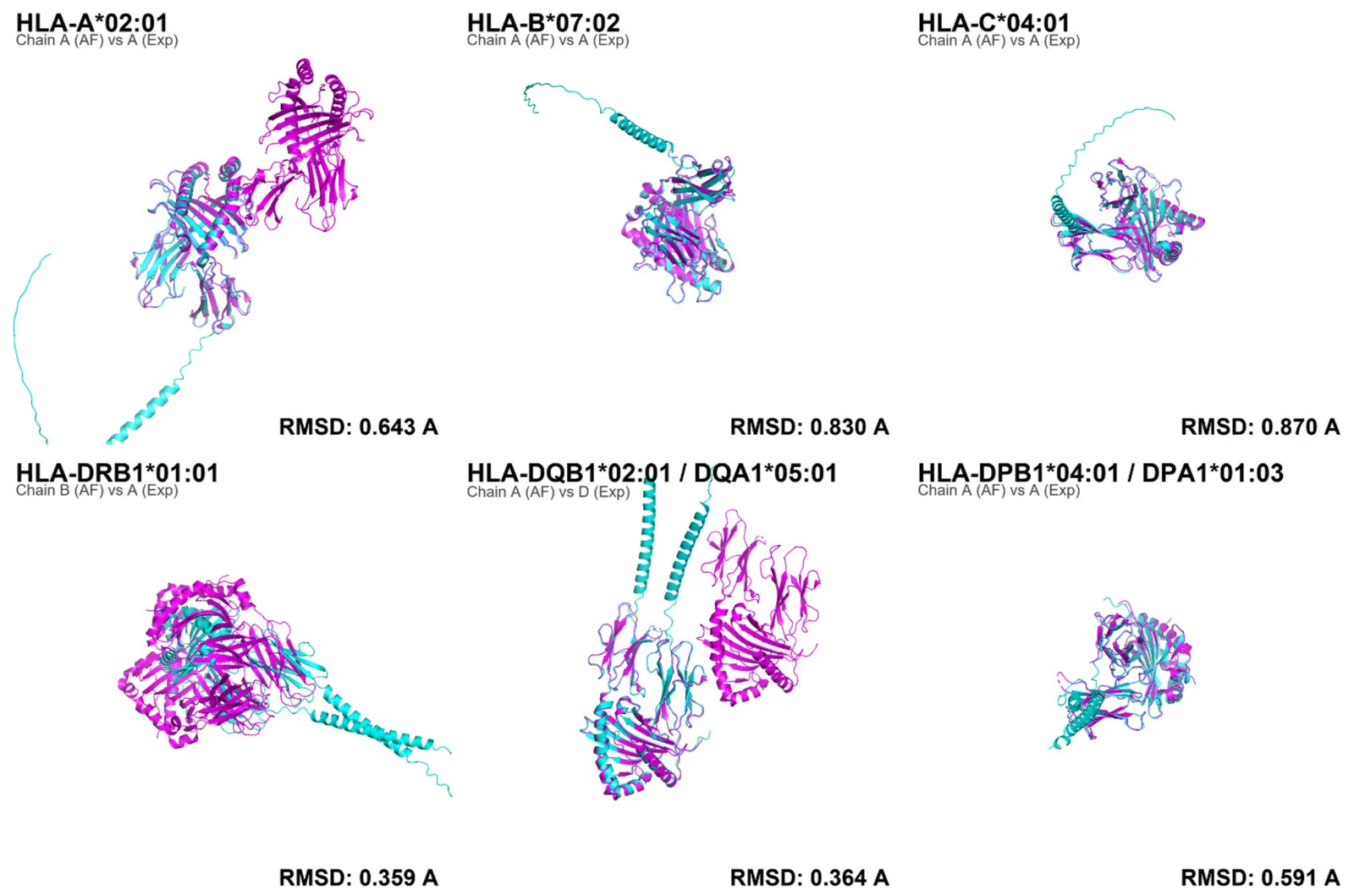
Structural validation of representative AlphaFold 3-predicted HLA models across all six loci. Enlarged superimposition of 6 representative AlphaFold 3 models (cyan; one per locus: HLA-A*02:01, HLA-B*07:02, HLA-C*04:01 for class I, and HLA-DRB1*01:01, HLA-DQB1*02:01/DQA1*05:01, HLA-DPB1*04:01/DPA1*01:03 for class II) onto their corresponding high-resolution PDB reference structures (purple). The complete 13-structure benchmark overlay is provided in Supplementary Figure S2. Unrounded Cα RMSD values (in Å) generated directly by PyMOL (version 3.1.6.1) are displayed on each panel (rounded to two decimal places in Table 1), confirming high structural fidelity across the folded extracellular domains. Abbreviations: HLA, Human Leukocyte Antigen; PDB, Protein Data Bank; RMSD, Root Mean Square Deviation.

Structural validation involved superimposition of 13 generated models (7 class I and 6 class II) against high-resolution crystalline structures available in the Protein Data Bank (PDB). Alignment revealed high conformational fidelity across both the 6 representative loci shown in Figure 1 and the complete 13-allele benchmark set shown in Supplementary Figure S2. The mean root mean square deviation (RMSD) for Cα atoms across all 13 tested alleles was 0.56 ± 0.20 Å (0.68 ± 0.19 Å for class I and 0.41 ± 0.09 Å for class II; Table 1). Across the 13 benchmarked complexes, full-length model pLDDT correlated inversely with experimental Cα RMSD (Spearman’s ρ = −0.560, *p* = 0.0463; Pearson’s r = −0.561, *p* = 0.0462), indicating that higher intrinsic AlphaFold 3 local confidence tracks with lower atomic deviation from X-ray crystal coordinates (for example, within class I, HLA-B*51:01 exhibited both the highest pLDDT [87.41] and the lowest RMSD [0.29 Å], whereas within class II, HLA-DPA1*01/DPB1*04 showed both the lowest ipTM [0.76] and the highest RMSD [0.59 Å]; Table 1). Importantly, because experimental PDB reference structures comprise exclusively truncated extracellular ectodomains, PyMOL Cα superimposition evaluates only the folded extracellular core and automatically excludes the unaligned, disordered transmembrane and cytoplasmic tails (which lower the global sequence-averaged pLDDT score to ~85–87 but do not enter the RMSD calculation). Instead, the slightly higher Cα RMSD observed in class I models (0.64–0.87 Å, except HLA-B*51:01 at 0.29 Å) compared to class II heterodimers (0.36–0.59 Å) arises from structural variations within the folded domains themselves: specifically, minor hinge-angle flexibility between the membrane-proximal α3 domain/non-covalently associated β2-microglobulin subunit and the α1/α2 peptide-binding platform, alongside subtle backbone relaxation of the α1/α2 helical walls in our peptide-free models relative to peptide-loaded crystal lattices. As detailed in Table 1, Cα deviation remained below 0.90 Å for all 13 reference structures, confirming near-native tertiary geometry suitable for solvent accessibility profiling.

### 2.2. Comparative Analysis of Eplet Mismatch Load

To assess the impact of 3D structure and physicochemical properties on theoretical antigenicity, we performed a comparative analysis of the distribution of candidate eplet mismatches across 10,000 simulated donor–recipient pairs using three methodologies: standard (HLAMM), verified (only experimentally verified), and our novel dual-filtered structural–biophysical approach (AlphaFold 3).

**Figure 2:**
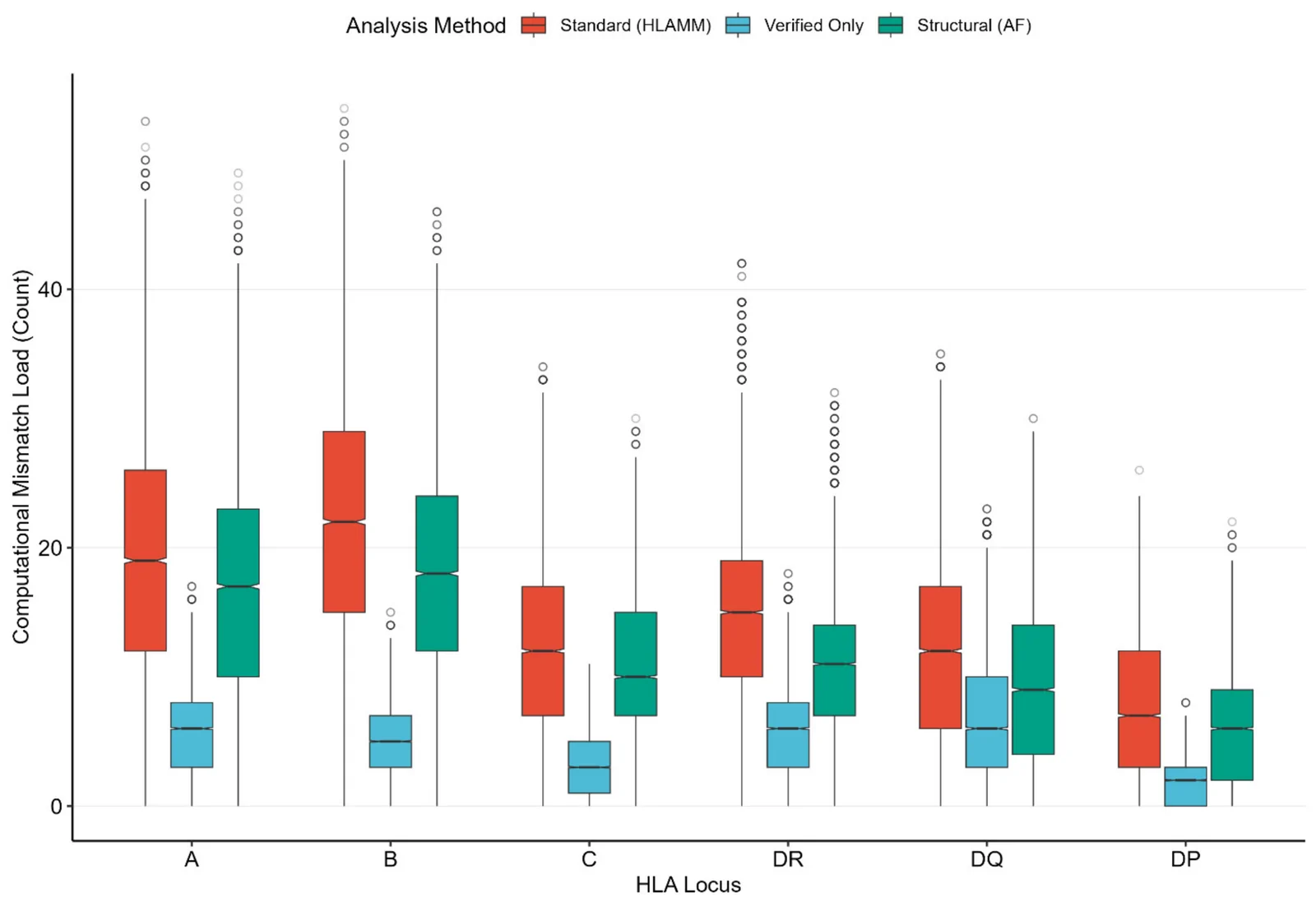
Structural and biophysical filtration alters computational mismatch load compared to standard algorithms (n = 10,000 donor–recipient pairs per locus). Boxplots display the median (horizontal center line) and interquartile range (IQR, box bounds from Q1 to Q3), with whiskers extending to 1.5 × IQR and outliers plotted as individual points. Because the Structural (AlphaFold 3, green) and Verified Only (light blue) methods represent sequential mathematical subsets of the baseline Standard (HLAMM, red) repertoire, the directional reduction in count (Standard ≥ Structural ≥ Verified) is a deterministic property of the filtration pipeline. Across the cohort, median [Q1; Q3] eplet counts for Standard (HLAMM), Structural (AlphaFold 3), and Verified Only were HLA-A: 19 [12; 26], 17 [10; 23], and 6 [3; 8]; HLA-B: 22 [15; 29], 18 [12; 24], and 5 [3; 7]; HLA-C: 12 [7; 17], 10 [7; 15], and 3 [1; 5]; HLA-DR: 15 [10; 19], 11 [7; 14], and 6 [3; 8]; HLA-DQ: 12 [6; 17], 9 [4; 14], and 6 [3; 10]; and HLA-DP: 7 [3; 12], 6 [2; 9], and 2 [0; 3] (see also (Supplementary Table S1)).

Because the dual-filter and verified-only algorithms operate by applying sequential exclusion criteria to the baseline HLAMM repertoire, they generate mathematical subsets of the standard eplet pool. Consequently, the directional reduction in mismatch counts (Standard ≥ Structural ≥ Verified) is an inherent deterministic property of the filtration algorithm rather than a stochastic difference requiring hypothesis testing (Figure 2). Comparing the descriptive distributions across all HLA loci demonstrates that the choice of computational framework substantially alters the quantified degree of molecular incompatibility.

#### 2.2.1. Reduction in Computational Mismatch Load

Application of the dual structural–biophysical filter resulted in a systematic reduction in the number of counted polymorphic residues compared to the standard HLAMM algorithm. This computational decrease reflects the algorithmic exclusion of residues classified as sterically inaccessible or below established empirical biophysical thresholds.

Class I: The most pronounced reduction was observed in the HLA-B locus, where the median load decreased from 22 [15; 29] (HLAMM) to 18 [12; 24] eplets. For the HLA-A locus, the median decreased from 19 [12; 26] to 17 [10; 23], and for HLA-C, from 12 [7; 17] to 10 [7; 15].

Class II: The “structural denoising” effect was also prominent across heterodimeric loci. In the HLA-DR locus, the median number of mismatches decreased from 15 [10; 19] to 11 [7; 14]; in the HLA-DQ locus, from 12 [6; 17] to 9 [4; 14]; and in the HLA-DP locus, from 7 [3; 12] to 6 [2; 9].

#### 2.2.2. Intermediate Positioning of the Dual-Filter Model

Metrics derived from our dual-filtered AlphaFold 3 approach consistently occupy an intermediate mathematical position between the standard HLAMM repertoire and the verified-only subset (Table 2). Crucially, because our biophysical gate (5th percentile of |*Q*_net_| and *H*_total_) functions strictly as an empirical exclusion filter calibrated on known antibody-verified epitopes (*N* = 170) rather than an independent predictor, this intermediate load is a partially circular consequence of model calibration rather than an independent validation of clinical accuracy.

For example, for the HLA-DR locus, the distribution was: HLAMM (15 [10; 19]) > AlphaFold (11 [7; 14]) > Verified (6 [3; 8]). This demonstrates that the dual-filter mathematically restricts the theoretical pool by screening out sterically hindered and sub-threshold interactions, leaving a subset of structurally accessible and biophysically reactive targets that have not yet been clinically verified. Ablation analysis of spatial (SASA-only) versus biophysical-only filtration: To determine whether the pronounced mismatch reduction in class II loci is driven genuinely by 3D structural steric hindrance rather than by the registry-calibrated 5th-percentile biophysical gate (|*Q_net_*| ≥ 0.3326 e and *H_total_* ≥ −7.265, derived from *N* = 170 verified eplets), we performed a five-arm component ablation study comparing (1) standard unfiltered HLAMM, (2) SASA-only (SASA ≥ 10.0 Å^2^), (3) biophysical-only (≥5th-percentile gate without SASA), (4) combined SASA + 5th-percentile gate, and (5) verified-only (Supplementary Table S9). Applying the Biophysical-only filter in isolation produced minimal reductions in class II mismatch counts: median loads remained unchanged at 7 [3; 12] for HLA-DP (eliminating a mean of only 0.25 eplets per pair; high-load reclassification of only 4.77%) and shifted by only one eplet to 14 [9; 19] for HLA-DR (mean 0.56 eplets removed; reclassification of 4.55%). Conversely, applying the SASA-only structural filter (SASA ≥ 10.0 Å^2^) accounted for 87.8% of the total combined eplet exclusion in both HLA-DR (removing a mean of 4.01 of 4.56 excluded eplets; median reduced from 15 [10; 19] to 11 [7; 14]; reclassification 25.99%) and HLA-DP (removing 1.79 of 2.04 excluded eplets; median reduced from 7 [3; 12] to 6 [2; 9]; reclassification 33.74%), as well as 58.2% to 69.1% of total exclusions across HLA-DQ (2.29 of 3.93 eplets) and class I loci (Supplementary Table S9). When the stringent 5th-percentile biophysical gate was layered on top of SASA (Combined SASA + 5th-percentile), median counts decreased by at most one additional eplet across class II loci (to 10 [6; 14] for HLA-DR, 8 [3; 12] for HLA-DQ, and 5 [2; 9] for HLA-DP, with combined reclassification proportions reaching 30.80%, 37.40%, and 38.34%, respectively). This ablation confirms that the downward shift in class II mismatch load is predominantly driven by 3D steric accessibility in the AlphaFold 3 models rather than by circular biophysical calibration.

### 2.3. Structural Deconvolution of Representative Class II Mismatches

**Figure 3:**
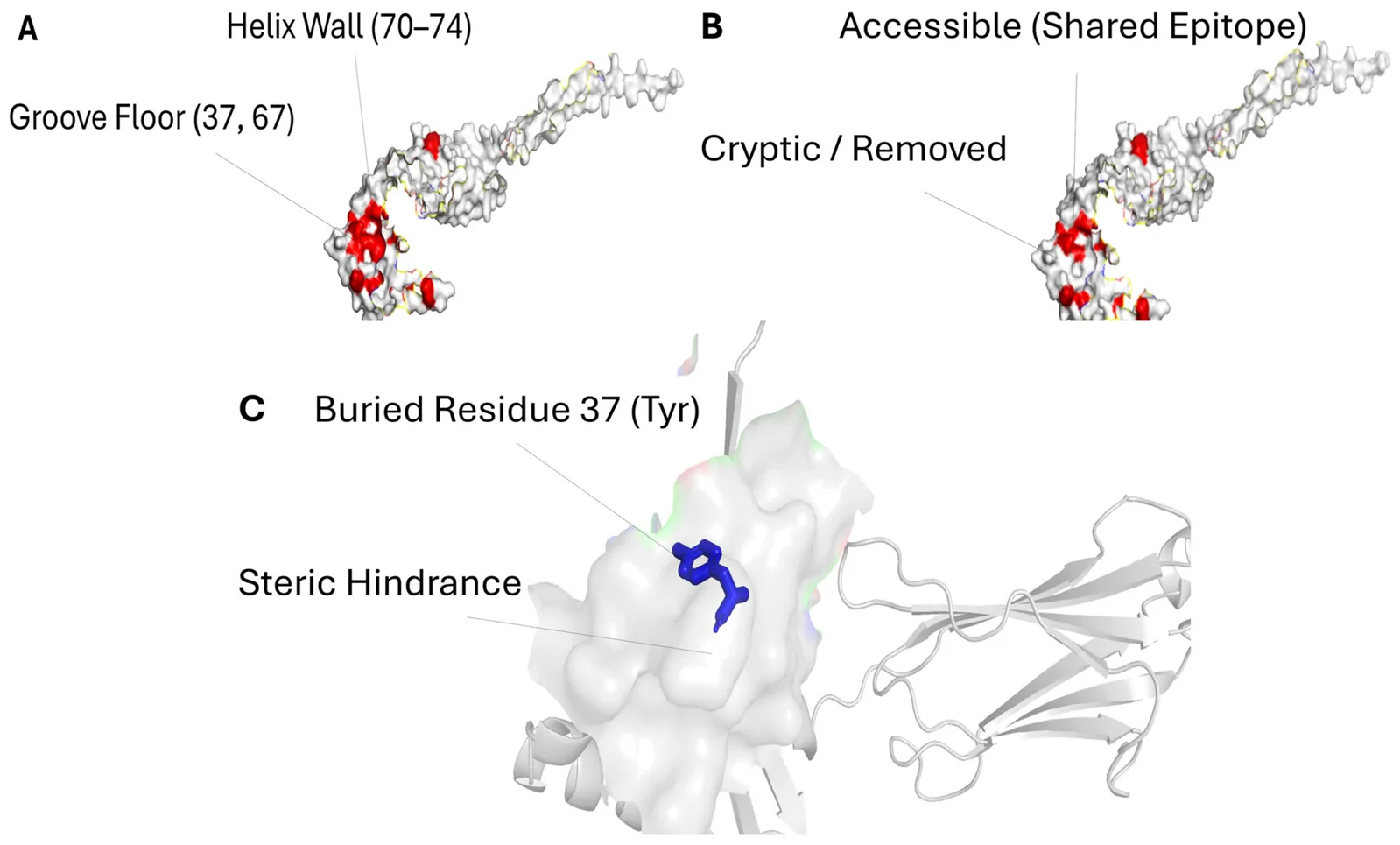
Structural re-evaluation of HLA-DR polymorphic residues in a high-risk donor–recipient pair (Donor DRB1*04:01/Recipient DRB1*07:01). The HLA-DRB1 chain is shown in a top-down viewing orientation into the peptide-binding cleft. (**A**) Baseline linear enumeration: Visualization of all 19 amino acid mismatches (red) identified by the standard HLAMM algorithm, which flags residues located on the floor of the peptide-binding groove (e.g., positions 37 and 67) as candidate targets. (**B**) Structural accessibility filter: Application of the quantitative SASA threshold (≥10.0 Å^2^ for retention; <10.0 Å^2^ excluded as white) removes sterically occluded residues located in deep pockets of the groove floor while retaining surface-exposed residues along the α-helical walls (red, e.g., positions 70–74). (**C**) Mechanism of steric occlusion: Magnified view of polymorphic residue Tyr37 (blue). Semi-transparent molecular surface rendering (gray, scaled to standard atomic radii) demonstrates that Tyr37 is sterically shielded by the surrounding α-helical walls of the native HLA tertiary structure (SASA < 10.0 Å^2^).

The observed distributional shifts indicate that standard linear methods yield higher baseline mismatch counts than 3D-filtered models by enumerating sterically buried or sub-threshold residues. To illustrate the structural basis of this algorithmic divergence, we performed a detailed structural deconvolution of a representative pair (Donor DRB1*04:01/Recipient DRB1*07:01). As demonstrated in Figure 3, the standard algorithm identifies 19 amino acid mismatches uniformly distributed throughout the molecule, including the floor of the peptide-binding groove (Figure 3A). However, the application of a SASA > 10 Å^2^ filter radically changes the candidate antigenic surface landscape.

Critical analysis reveals that residues located deep within the groove pockets (specifically Tyr37) fall below the quantitative 10.0 Å^2^ SASA threshold. Notably, because our complexes were modeled in a standardized peptide-free conformation, the computed SASA for groove-floor residues represents a conservative upper-bound estimate of solvent exposure: since Tyr37 is already sterically occluded by the α-helical walls of the empty HLA cleft (SASA < 10.0 Å^2^; Figure 3C), the presence of a bound peptide occupying the groove in vivo would impose even greater steric shielding, further precluding direct B-cell receptor contact.

Thus, our method reclassifies these “mismatches” as structurally inert, leaving only surface-exposed alpha-helix regions as valid targets, such as the “shared epitope” zone (Figure 3B). This visually explains the mechanism of eplet load reduction observed at the population level.

### 2.4. Biophysical and Electrostatic Analysis

The number of eplet mismatches serves as a baseline surrogate marker of theoretical antigenicity. Because antibody recognition also depends on local physicochemical complementarity, we conducted a comparative analysis of cumulative Henderson–Hasselbalch point-charge sums and Kyte–Doolittle hydropathy sums as first-order heuristic amino acid indices [17,18] for each simulated donor–recipient pair (noting that these sequence-derived sums do not represent continuum surface electrostatic potentials or true binding free energies).

#### 2.4.1. Cumulative Electrostatic Charge Index

**Figure 4:**
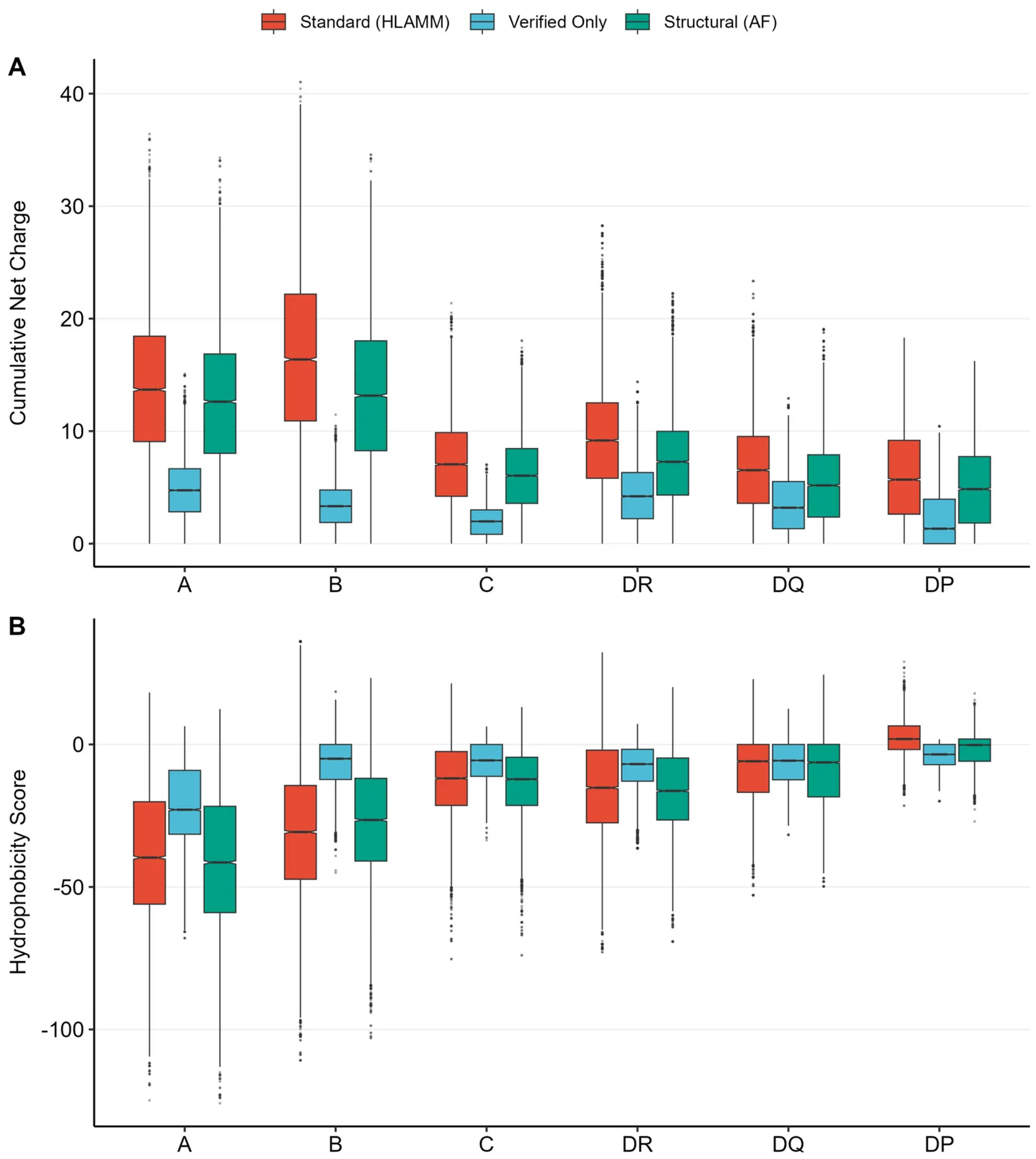
Structural correction of physicochemical properties of eplet load. (**A**) Cumulative absolute net charge (|Q_net_| at pH 7.4, in elementary charge units e). Standard linear enumeration (HLAMM, red) yields higher cumulative charge sums due to the inclusion of sterically occluded (SASA < 10.0 Å^2^) or sub-threshold ionizable residues. Sequential filtration (Structural AlphaFold 3, green) restricts summation to surface-accessible, suprathreshold candidate patches, shifting distributions toward Verified Only values (light blue). Median [Q1; Q3] cumulative charges for Standard, Structural, and Verified Only were: HLA-A: 13.69 [9.07; 18.44], 12.62 [8.03; 16.86], and 4.74 [2.83; 6.66]; HLA-B: 16.37 [10.91; 22.18], 13.10 [8.23; 17.93], and 3.33 [1.88; 4.77]; HLA-C: 7.05 [4.21; 9.87], 6.03 [3.58; 8.43], and 1.98 [0.83; 3.00]; HLA-DR: 9.17 [5.81; 12.52], 7.28 [4.32; 9.98], and 4.21 [2.22; 6.32]; HLA-DQ: 6.53 [3.58; 9.53], 5.14 [2.36; 7.67], and 3.19 [1.33; 5.52]; and HLA-DP: 5.69 [2.62; 9.18], 4.85 [1.83; 7.74], and 1.33 [0.00; 3.95]. (**B**) Cumulative Kyte–Doolittle hydropathy score (H_total_). Median [Q1; Q3] hydropathy scores for Standard, Structural, and Verified Only were: HLA-A: −39.70 [−56.02; −20.10], −41.40 [−59.00; −21.70], and −22.90 [−31.50; −9.10]; HLA-B: −30.70 [−47.30; −14.40], −26.10 [−39.40; −11.90], and −5.00 [−12.30; 0.00]; HLA-C: −11.90 [−21.40; −2.50], −10.50 [−20.40; −3.50], and −5.60 [−11.20; 0.00]; HLA-DR: −15.20 [−27.50; −2.00], −16.30 [−26.50; −4.78], and −6.90 [−12.90; −1.70]; HLA-DQ: −5.90 [−16.80; 0.00], −5.50 [−16.60; 0.50], and −5.70 [−12.40; 0.00]; and HLA-DP: 1.90 [−1.80; 6.50], −0.20 [−5.90; 1.90], and −3.50 [−7.10; 0.00] (paired Hodges-Lehmann median differences and rank-biserial effect sizes are provided in Table 3 and Supplementary Table S1).

Our analysis demonstrated that the standard HLAMM algorithm yields higher cumulative absolute charge scores compared to the dual-filtered model across all loci (Figure 4A). Because the dual-filter excludes sterically buried and sub-threshold residues from the summation of non-negative absolute charges (|*Q_net_*|), this directional decrease is a direct mathematical consequence of the filtration pipeline (rank-biserial r ranging from 0.807 to 0.863; Table 3). Crucially, rather than being buried within a hydrophobic protein interior, these excluded ionizable residues are predominantly located along the β-sheet floor and deep within the peptide-anchoring pockets (P1, P4, P6, P7, and P9) of the HLA peptide-binding cleft or at the inter-chain α/β and heavy chain–*β*_2_m interface. This topological localization is directly confirmed by experimental X-ray crystallographic studies of the benchmark structures in Table 1 [19,20,21,22,23]: specifically, crystallographic analyses of HLA class I [23], HLA-DR1 (PDB: 1DLH) [22], HLA-DQ8 (PDB: 1JK8) [21], HLA-DQ2 (PDB: 1S9V) [20], and HLA-DP2 (PDB: 3LQZ) [19] demonstrate that polymorphic charged residues—such as groove-floor acidic/basic triads (e.g., βGlu26/βGlu68 in HLA-DP [19], βLys71 and pocket-lining Arg/Glu/His residues in HLA-DR and HLA-DQ [20,21,22], or pocket-anchoring Asp/Glu/Arg residues in class I [23]) and conserved inter-chain salt-bridge partners (e.g., βAsp57 pairing with αArg76 in pocket P9 [20,21])—serve to coordinate bound peptide side chains or stabilize the cleft architecture while exhibiting low solvent accessibility (SASA < 10.0 Å^2^) that sterically precludes direct contact with an antibody complementarity-determining region (CDR) loop.

#### 2.4.2. Cumulative Hydropathy Index

In contrast to cumulative charge, distributional shifts in signed Kyte–Doolittle hydropathy sums were highly locus-dependent (Figure 4B and Table 3). Because Kyte–Doolittle hydropathy spans both negative (hydrophilic) and positive (hydrophobic) values, removing buried or sub-threshold residues produced negligible to modest distributional shifts for HLA-DR (*r* = 0.069; Hodges–Lehmann Δ median = 0.55 [0.40; 0.70]), HLA-C (*r* = 0.122; Δ median = −0.55 [−0.75; −0.45]), and HLA-DQ (r = 0.180; Δ median = −1.90 [−2.05; −1.70]), and moderate shifts for HLA-A (*r* = 0.472) and HLA-B (*r* = 0.516). The largest hydropathy shift occurred at the HLA-DP locus (*r* = 0.689; Δ median = 4.70 [4.65; 4.85]), where the standard HLAMM algorithm yielded a positive median hydropathy sum (1.90 [−1.80; 6.50]), whereas the dual-filter shifted the median to near-neutral levels (−0.20 [−5.90; 1.90]) due to the exclusion of buried hydrophobic residues.

#### 2.4.3. Magnitude of Algorithmic Shifts

Quantification of the paired distributional shifts using Hodges–Lehmann median differences and rank-biserial effect sizes (*r*) characterize the degree of algorithmic divergence across the three computational definitions (Table 3). Rather than defining separate immunological populations, the dual-filter isolates a restricted mathematical subset of candidate eplets that satisfy spatial accessibility and empirical amino acid index criteria [17,18].

### 2.5. Fixed-Cutoff Categorization and Physicochemical Covariance

To illustrate how sequential structural and biophysical filtering alters categorical mismatch distributions, we evaluated the proportion of simulated donor–recipient pairs crossing published numerical eplet cutoffs. For baseline categorization, we applied conventional literature thresholds [2,3], grouping simulated pairs into ≥10 eplets (high load), 1–9 eplets (intermediate/low load), and 0 eplets (full match). Because these published cutoffs were empirically calibrated for unfiltered 1D HLAMM counts, applying them to a restricted mathematical subset of spatially accessible and biophysically suprathreshold candidate eplets serves strictly as an exploratory demonstration of algorithmic divergence rather than a validated reclassification of clinical risk.

#### 2.5.1. Fixed-Threshold Categorization of High-Load Simulated Pairs

**Figure 5:**
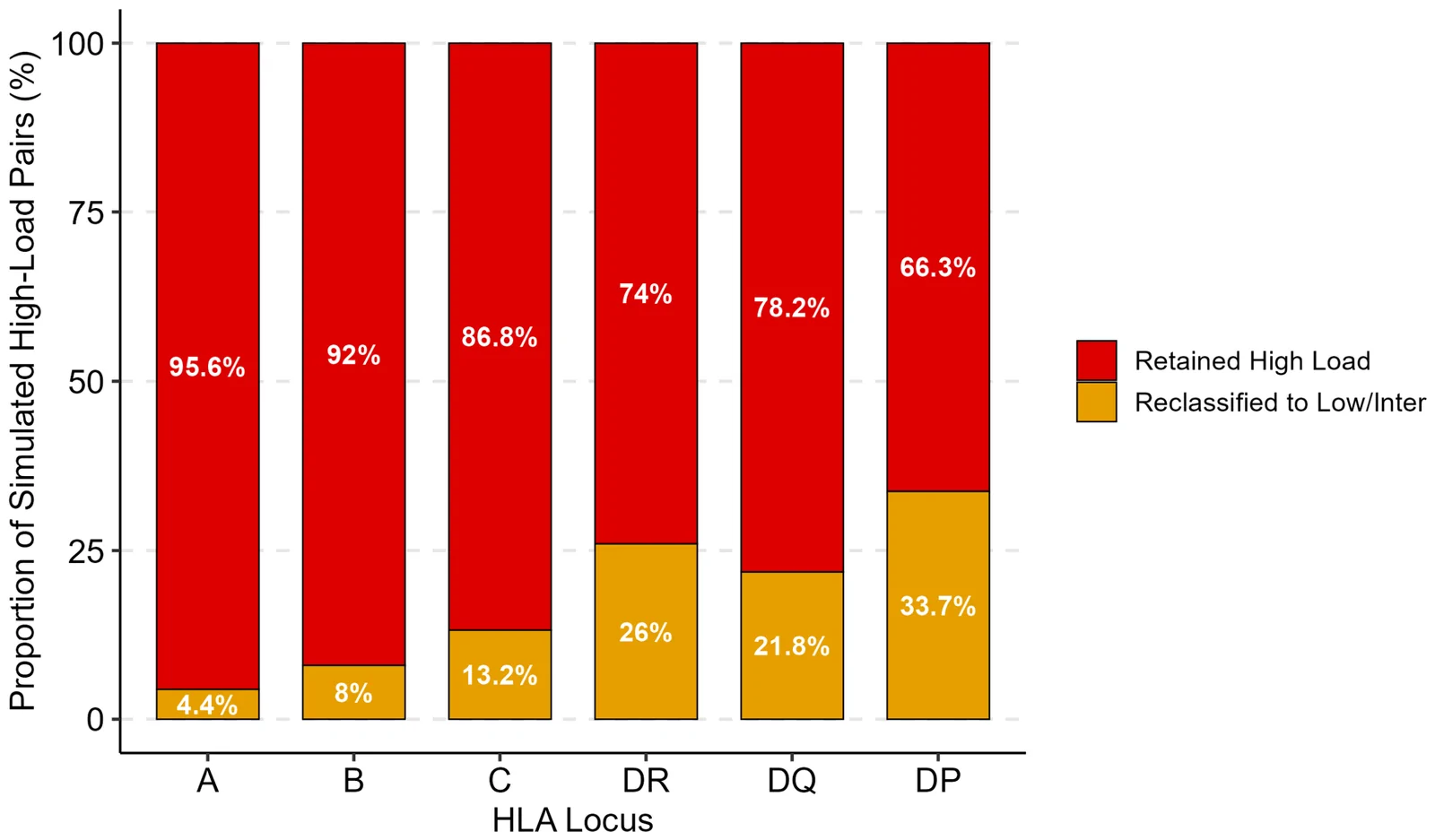
AlphaFold-based dual-filter reclassification of simulated high-load donor–recipient pairs. Stacked bar charts illustrate the proportion of simulated donor–recipient pairs initially categorized as high load (≥10 eplets by standard linear counting) that either retain high computational load (≥10 eplets, red) or cross below the threshold into the intermediate/low load category (1–9 eplets, orange) after applying the structure-based dual-filter model. This illustrates the divergence in computational categorization for class II alleles when predicted sterically cryptic or biophysically sub-threshold residues are excluded from the baseline sequence count. No simulated pair was reclassified into the “full match” (0 eplets) category.

To demonstrate how structural filtration affects fixed threshold categorization, we evaluated the subset of simulated donor–recipient pairs initially exceeding the ≥10-eplet cutoff under the standard HLAMM algorithm (*n* = 8128 for HLA-A, *n* = 8889 for HLA-B, *n* = 6532 for HLA-C, *n* = 7718 for HLA-DR, *n* = 6006 for HLA-DQ, and *n* = 3693 for HLA-DP; Figure 5 and Supplementary Table S4). For class I loci, structural modeling demonstrated high concordance with baseline linear counts: only 4.42% (359/8128; 95% CI: 3.99–4.89%) of HLA-A, 7.95% (707/8889; 95% CI: 7.41–8.53%) of HLA-B, and 13.24% (865/6532; 95% CI: 12.44–14.09%) of HLA-C high-load pairs dropped below the 10-eplet cutoff after filtration. This indicates that the majority of eplet-defining polymorphic residues in class I molecules are surface-exposed and biophysically suprathreshold. In contrast, class II high-load subsets exhibited a larger proportion of pairs crossing below the 10-eplet benchmark cutoff. Application of the structural–biophysical filter shifted 21.84% (1312/6006; 95% CI: 20.82–22.91%) of high-load pairs for HLA-DQ and 25.99% (2006/7718; 95% CI: 25.02–26.98%) for HLA-DR into the 1–9 eplet category. For HLA-DP—where the full-cohort baseline median is 7 [3; 12] and therefore only 36.93% (3693/10,000) of pairs initially have ≥10 eplets—33.74% (1246/3693; 95% CI: 32.23–35.28%) of that high-load subset dropped below the ≥10-eplet cutoff after filtration (corresponding to 12.46% of the total 10,000-pair cohort). Rather than representing validated false-positive clinical risk, these percentages strictly quantify the proportion of high-load pairs whose computed count crosses a fixed linear threshold following structural and biophysical filtering. We performed an additional quantitative assessment of categorical agreement across the fixed numerical cutoffs (0, 1–9, and ≥10 eplets) between the two methods using Cohen’s weighted kappa coefficient (*κ_w_*). Because *κ_w_* assumes independent observations, we computed two-sided 95% confidence intervals across the full population-weighted cohort (*n* = 10,000), the strictly deduplicated unique biological donor–recipient diploid genotype pairs (nunique), and an allele-level cluster bootstrap treating the structural allele library as the unit of uncertainty (Supplementary Tables S5 and S7). All three evaluations confirmed the exact same locus-dependent dichotomy: HLA-A (*κ_w_* = 0.948 [95% CI: 0.943–0.954]; *κ_w_*_,unique_ = 0.884 [0.864–0.905]), HLA-B (*κ_w_* = 0.835 [0.821–0.849]; *κ_w_*_,unique_ = 0.831 [0.809–0.853]), HLA-C (*κ_w_* = 0.879 [0.870–0.887]; *κ_w_*_,unique_ = 0.851 [0.835–0.868]), and HLA-DQ (*κ_w_* = 0.857 [0.849–0.865]; *κ_w_*_,unique_ = 0.824 [0.804–0.844]) consistently demonstrated “almost perfect” agreement (*κ_w_* > 0.80), whereas HLA-DR (*κ_w_* = 0.715 [0.701–0.729]; *κ_w_*_,unique_ = 0.731 [0.700–0.761]) and HLA-DP (*κ_w_* = 0.789 [0.778–0.799]; *κ_w_*_,unique_ = 0.748 [0.731–0.765]) showed only “substantial” agreement (*κ_w_* < 0.80). Similarly, non-parametric allele-level cluster bootstrap confidence intervals (1000 iterations; Supplementary Table S5) preserved this separation, with bootstrap *κ_w_* 95% CIs of 0.846–0.972 for HLA-A, 0.788–0.884 for HLA-B, 0.849–0.896 for HLA-C, and 0.783–0.891 for HLA-DQ, compared to 0.661–0.768 for HLA-DR and 0.740–0.817 for HLA-DP.

This statistical discrepancy underscores that the integration of biophysical constraints with structural filtration reveals a distinct dimension of class II heterodimer antigenicity not captured by standard linear eplet counting, particularly for the highly polymorphic DR and DP loci.

#### 2.5.2. Covariance of Electrostatic Charge and Hydropathy

To elucidate the biophysical basis of this locus-specific divergence, we investigated whether structural eplet count serves as a consistent proxy for cumulative electrostatic charge and hydropathy.

**Figure 6:**
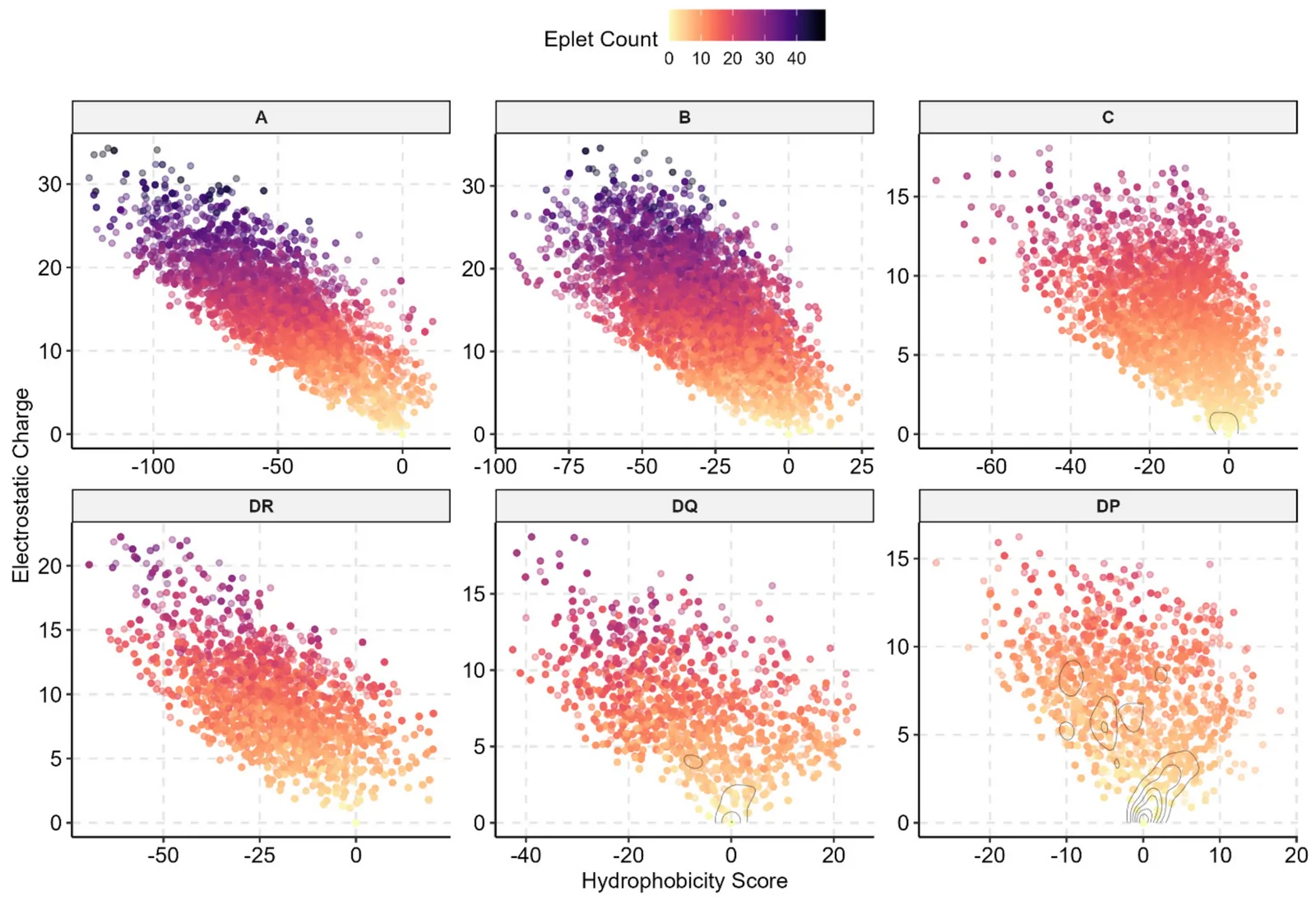
Physicochemical landscape of HLA mismatches. Scatter plots illustrate the relationship between total electrostatic charge (*Y*-axis) and hydrophobicity (*X*-axis) for structural mismatches across six HLA loci. Each point represents a donor–recipient pair. We observed a strong negative correlation between charge and hydrophobicity for most loci; most pronounced for HLA-A (*ρ* = −0.842) and HLA-B (*ρ* = −0.692). HLA-C (*ρ* = −0.563), HLA-DR (*ρ* = −0.645), and HLA-DQ (*ρ* = −0.578) also demonstrated a high degree of association (*p <* 0.0001 for all), consistent with the hydrophilic nature of charged residues. In contrast, HLA-DP stands out with a unique profile showing the weakest correlation (*ρ* = −0.400; *p* < 0.0001), indicating that electrostatic potential and hydrophobicity act as more independent drivers of theoretical antigenicity specifically at this locus. This underscores the necessity for multidimensional profiling, especially for accurate risk assessment of HLA-DP. Data represent *n* = 10,000 simulated European donor–recipient pairs sampled from 76 high-frequency alleles and *α*/*β* haplotypes (population frequency ≥1.0%: 14 HLA-A, 14 HLA-B, 13 HLA-C, 10 HLA-DR, 10 HLA-DQ, and 15 HLA-DP; Supplementary Table S13) within the 80-model AlphaFold 3 structural repository (Supplementary Table S6).

We found a strong, significant negative correlation between total electrostatic charge and hydrophobicity for most loci, particularly HLA-A (*ρ* = −0.842; *p* < 0.0001), HLA-B (*ρ* = −0.692; *p* < 0.0001), and HLA-C (*ρ* = −0.563; *p* < 0.0001) (Figure 6 and Supplementary Table S2). This reflects a fundamental biophysical principle: the accumulation of charged surface residues inevitably leads to increased hydrophilicity (Figure 6). For these loci, a high eplet load generally correctly signals high electrostatic and low hydrophobic potentials.

However, HLA-DP exhibited the weakest zero-order correlation between cumulative electrostatic charge and hydropathy scores (*ρ* = −0.400; *p* < 0.0001), whereas HLA-DQ and HLA-DR occupied an intermediate position (*ρ* = −0.578 and *ρ* = −0.645, *p* < 0.0001, respectively). Because HLA-DP also exhibits the lowest baseline mismatch count (structural median 6 [2; 9]) and a narrower numerical score range—which can statistically attenuate correlation coefficients via range restriction—we performed two control analyses: (1) a strict 1:1 count-distribution-matched subsampling across all six loci (*n* = 2861 pairs per locus matched exactly across mismatch counts 1–18; mean count = 10.37), and (2) partial Spearman rank correlations conditioned on mismatch count (Supplementary Table S8). In the 1:1 count-matched analysis, correlations attenuated moderately across high-load loci (*ρ*_matched_ = −0.663 for HLA-A, −0.588 for HLA-DR, −0.538 for HLA-B, −0.436 for HLA-DQ, and −0.358 for HLA-C; all *p* < 0.0001), confirming that range width contributes partially to the raw coefficients. Nevertheless, HLA-DP retained the weakest charge–hydropathy correlation even under identical count distributions (*ρ*_matched_ = −0.268; *p* = 2.91 × 10^−48^). Conditioning directly on mismatch count revealed that while zero-order correlations at class I and HLA-DQ loci are largely driven by cumulative count scaling (*ρ*_partial_ = −0.198 for HLA-A and −0.108 for HLA-DQ), the inverse association at HLA-DP (*ρ*_partial_ = −0.385; *p* < 0.0001) and HLA-DR (*ρ*_partial_ = −0.439; *p* < 0.0001) reflects intrinsic amino acid composition at fixed mismatch loads. Restricting the analysis strictly to deduplicated unique diploid genotype pairs (*n*_unique_; Supplementary Table S7) yielded virtually identical correlations, with HLA-DP again showing the weakest charge–hydropathy association (*ρ*_unique_ = −0.339, *p* = 3.44 × 10^−124^) and count–hydropathy association (*ρ*_unique_ = −0.209, *p* = 9.99 × 10^−47^) across all six loci. We also examined the mathematical relationship between total eplet count and the cumulative physicochemical indices (Supplementary Figure S1 and Table S3). Because cumulative charge ($Q_{\mathrm{total}}=\sum |Q_{\mathrm{net}}|$) is defined as the sum of non-negative absolute charges (|*Q*_net_|) across all mismatched eplets (including residues shared across overlapping eplets), the strong positive correlation between eplet count and cumulative charge across all loci (Spearman’s *ρ* range 0.926–0.978) is an expected algebraic consequence of additive summation rather than an independent biological validation of eplet count. In contrast, because Kyte–Doolittle hydropathy is a signed quantity (summing both hydrophilic and hydrophobic residues), eplet count was only weakly associated with cumulative hydropathy at the HLA-DP locus both in the full cohort (ρ = −0.292; *p* < 0.0001; Supplementary Figure S1) and in the 1:1 count-matched subsample (*ρ_matched_* = −0.138; *p* = 1.33 × 10^−13^, compared to *ρ_matched_* = −0.656 for HLA-A). This indicates that at the HLA-DP locus, signed hydropathy sums vary largely independently of total eplet count, as uncharged hydrophobic and hydrophilic substitutions can offset or accumulate without proportional changes in total mismatch number.

## 3. Discussion

The evaluation of histocompatibility in solid organ transplantation has traditionally relied on linear assessments of amino-acid polymorphisms, a conceptual framework established by the foundational HLAMM algorithm [1,24]. Although this represented a transformative advancement over serological typing, the approach implicitly assumes that all polymorphic residues are equally immunologically accessible. Our findings demonstrate that this simplification introduces computational noise, yielding higher baseline mismatch counts than 3D-filtered models. By integrating the high-fidelity structural capabilities of AlphaFold 3 [15] into the compatibility pipeline, we achieved a pronounced “structural denoising” effect, enabling the separation of spatially accessible, biophysically suprathreshold candidate eplets from cryptic, sterically occluded residues. These findings support the development of an exploratory structural-filtering framework, highlighting the critical need for harmonization across quantitative counting-based models and qualitative 3D-structural–biophysical approaches. This conceptual transition aligns directly with the differential immunogenicity model emphasized by Tambur and colleagues, who demonstrated that individual eplets do not contribute equally to allosensitization risk and instead form a qualitative hierarchy [13]. A simple linear count implicitly assumes equivalence among all mismatched residues, an assumption that contradicts this empirical clinical hierarchy [13]. Our structural and biophysical pipeline provides a mechanistic rationale for differential immunogenicity: eplets that are sterically cryptic or lack sufficient electrostatic and hydrophobic potential are computationally deprioritized, whereas highly accessible and thermodynamically reactive surface patches are retained. Consequently, integrating 3D structural parameters offers a physical framework that complements and mathematically supports the empirically observed hierarchy of eplet immunogenicity.

The mechanistic basis for the observed risk reclassification lies in the inherent topological limitations of linear sequencing. Standard algorithms operate on the premise that any amino acid substitution constitutes a potential target, disregarding steric shielding by the native tertiary structure of the HLA complex. By applying a dual-filter approach—combining strict SASA thresholds using the Shrake–Rupley algorithm [25,26] with empirical biophysical cutoffs derived from verified eplets—our model filters out not only “cryptic” mismatches located deep within the peptide-binding groove, but also sterically exposed patches that lack the thermodynamic potential (charge or hydrophobicity) to sustain antibody binding. This approach aligns with the fundamental structural biology principle that B-cell receptors require a solvent-exposed and biophysically reactive interaction interface to initiate binding, as originally postulated by Barlow et al. [27]. Consequently, the “structural denoising” strategy achieved in our study effectively bridges the gap between theoretical antigenicity and 3D-structural antigenicity, addressing a critical distinction for optimal epitope matching highlighted by Kramer et al. [8].

Our findings highlight a striking divergence in method performance between HLA classes. For HLA class I molecules, the standard linear algorithm demonstrated high concordance with structural modeling, likely due to the relative conformational rigidity of the heavy chain-*β*_2_m complex [23]. However, for the heterodimeric HLA class II molecules, standard methods assigned a “high-risk” status to approximately one-third of the cohort that structural analysis reclassified as lower risk. This observation highlights a theoretical divergence that may intersect with current risk stratification models, which often link high molecular mismatch loads with adverse outcomes [2]. The potential clinical translation of this computational class II reclassification must strictly distinguish between pre-transplant donor selection and post-transplant immunosuppression management, as these decisions carry fundamentally different risk tolerances and evidence requirements. In the context of donor selection, integrating structural profiling could potentially expand the candidate donor pool by theoretically reclassifying specific high-load class II mismatches into lower computational load categories [3], although prospective clinical validation against DSA formation and graft survival is required before any clinical implementation. Conversely, the application of computational profiling for immunosuppression minimization (e.g., lowering tacrolimus trough levels) poses severe clinical risks, including under-immunosuppression and subsequent graft loss [2,9]. While our structural data suggest that many HLA-DR, -DQ, and -DP mismatches possess a significantly lower theoretical antigenic load than predicted by linear sequence counting, applying this framework to reduce maintenance therapy demands a substantially higher threshold of clinical evidence. Such post-transplant interventions cannot rely solely on in silico metrics; they must be rigorously evaluated in prospective clinical trials with defined immunological endpoints (e.g., de novo DSA formation) before any structural-based adjustment to immunosuppressive protocols can be considered [8,9].

A major distinction of our pipeline is the exclusive utilization of AlphaFold 3 [15] for full-length complex generation, bypassing the traditional reliance on X-ray crystallography databases (e.g., PDB [28]). While previous structural algorithms like Snowflake [11] utilized a hybrid dataset of existing PDB crystals and isolated docking simulations, such approaches suffer from methodological heterogeneity and critical scalability limits—especially given the extreme polymorphism of the HLA system (>35,000 known alleles [29]). By employing AlphaFold 3 to natively co-model the full-length heterodimeric complexes (heavy chain with *β*_2_-microglobulin for class I, and *α*/*β* chains for class II) in a standardized peptide-free conformation, we ensure a uniform, high-throughput structural dataset free from heterogeneous crystallographic artifacts. This de novo generation capability democratizes structural histocompatibility, proving that physical crystallization is no longer a prerequisite for highly accurate, allele-specific antigenic profiling.

Importantly, steric accessibility represents only the initial prerequisite for antigen recognition, whereas antibody binding affinity is governed by local surface complementarity and physicochemical interactions. Consequently, a comprehensive compatibility assessment must extend beyond counting “visible” eplets to quantifying their physicochemical properties, specifically electrostatic charge and hydropathy [17,18,30]. Our comparative analysis identified a clear divergence between histocompatibility classes. HLA class I molecules exhibited a highly conserved profile in which structural eplet load strongly tracked cumulative physicochemical scores, consistent with the structural uniformity of the heavy chain–*β*_2_m complex [23]. In contrast, the HLA-DP locus demonstrated a weakened covariance between heuristic charge and hydropathy scores. Although part of the difference in zero-order correlation coefficients across loci is attributable to the narrower mismatch count range of HLA-DP, our 1:1 count-distribution-matched subsampling and partial correlation analyses confirmed that HLA-DP retains the weakest association between eplet count and hydropathy (*ρ*_matched_ = −0.138) as well as between charge and hydropathy (*ρ*_matched_ = −0.268) even when evaluated at identical mismatch loads. Within HLA class II heterodimers, discrete substitutions in hypervariable regions can introduce either uncharged hydrophobic motifs or disproportionately high local electrostatic charges despite low total eplet counts—a finding that echoes the work of Amaya-Ramirez et al. [12], who demonstrated the superiority of 3D-surface patch descriptors over linear sequence enumeration. However, the clinical context of these locus-specific findings must be carefully delineated. While our pipeline demonstrated the most pronounced downward computational risk reclassification for the HLA-DP locus (33.7%), it is critical to note that HLA-DP mismatch loads are not routinely incorporated into mainstream clinical algorithms for immunosuppression management or tacrolimus-trough targeting (such as those described by Wiebe et al.) [2,3]. Therefore, while the structural and biophysical profiling of HLA-DP provides valuable theoretical insights into its distinct physicochemical profile, its direct clinical relevance remains limited in current standard practice until its role in allosensitization is more definitively integrated into future multiparametric clinical models.

While our study provides a comprehensive structural framework, several limitations must be acknowledged. First, our pre-computed structural repository encompasses 80 full-length HLA complexes, from which 76 high-frequency European allotypes and *α*/*β* haplotypes (frequency ≥ 1.0%) were used to simulate the virtual cohort of 10,000 donor–recipient pairs. While this selection captures the clinically dominant eplets and critical hypervariable positions in the European demographic, it inherently excludes rare, structurally distinct alleles and non-European ethnicity-specific eplets found among the >35,000 catalogued variants [29].

Consequently, the quantitative population-level estimates reported in this study—including median mismatch loads and the proportions of high-risk reclassification across loci—are bounded by the genetic architecture of the European population model. Because HLA allele frequencies and haplotype structures vary across geographic and ethnic populations, divergent frequency distributions and linkage disequilibrium patterns in non-European cohorts may alter both the baseline distribution of theoretical mismatches and the specific functional *α*/*β* chain pairings in heterodimeric HLA-DQ and HLA-DP complexes, potentially modifying interface geometry and surface accessibility profiles. Therefore, although the computational structural–biophysical filtration pipeline itself is population-agnostic, its application to other populations requires expanding the pre-computed AlphaFold 3 structural library to incorporate ancestry-specific allotypes and recalibrating simulation parameters using population-matched haplotype frequencies.

Second, our analysis relied on static, peptide-free structural predictions generated by AlphaFold 3 [15]. While modeling empty clefts provides a rigorous conservative upper-bound SASA estimate for groove-floor residues already occluded by the *α*-helical walls (such as Tyr37), static peptide-free models do not capture conformational plasticity (‘breathing’) or peptide-induced steric shielding of groove-adjacent side chains upon specific peptide loading [12].

Third, our in silico approach does not model physiological variables that dictate in vivo accessibility, such as glycosylation patterns, membrane proximity, or the specific docking orientation of the B-cell receptor.

Fourth, our pipeline strictly evaluates structural and physicochemical parameters, omitting expression-linked genetic variables. For instance, the immunogenicity of HLA-DP is heavily modulated by expression levels variant linked to the rs9277534 [31,32]. The lack of integration of such genomic expression data represents a limitation of the current framework and highlights a critical direction for future multiparametric risk models [33].

Fifth, the empirical biophysical thresholds applied in our dual-filter pipeline were derived directly from the verified eplet registry. Consequently, the finding that our structural model yields mismatch loads intermediate between standard linear counts and verified counts is partially circular by construction. This calibration establishes a theoretical minimum for antibody binding based on currently known epitopes, but it requires independent experimental datasets to validate its true predictive accuracy.

Sixth, relying on sequence-derived point charges and Kyte–Doolittle hydropathy sums presents an inherent biophysical limitation. As demonstrated by Blythe and Flower, single-scale amino acid characteristics perform poorly as independent predictors of antibody epitope location [34]. Our heuristic sums do not equate to exact electrostatic surface potentials (which require solving the Poisson–Boltzmann equation) or true binding free energies (ΔG). Specifically, applying standard intrinsic p*K_a_* values does not account for the local protein dielectric microenvironment, intramolecular hydrogen bonding, or salt bridges, which can shift protolytic equilibria. Although our initial spatial filter (SASA > 10 Å^2^) excludes deeply buried residues (where desolvation-induced p*K_a_* perturbations are most extreme), local surface electrostatics may still modulate the ionization state of functional groups with dissociation constants near physiological pH (most notably the imidazole group of histidine), potentially affecting the retention or exclusion of borderline eplets near the 5th-percentile threshold (see sensitivity analysis across His p*K_a_* 5.5–7.0 in Supplementary Table S12). While our dual-filter approach attempts to mitigate this by using these metrics strictly as negative exclusion thresholds combined with 3D structural (SASA) constraints, they remain surrogate approximations of thermodynamic binding strength.

Seventh, the risk categorization thresholds utilized in our comparative analysis (e.g., ≥10 eplets for high risk) were originally calibrated for conventional sequence-based algorithms [2,3]. Because the dual-filter approach generates a systematically smaller and distinct numerical metric, applying the same cutoffs carries inherent statistical limitations. Defining clinically accurate, structurally calibrated risk thresholds requires independent empirical validation against immunological outcomes—initially through retrospective re-analysis of well-characterized patient cohorts with longitudinal de novo DSA monitoring—analogous to the foundational calibration studies established for standard eplet models.

Eighth, while alternative 3D structural tools such as Snowflake [11], HLA-EpiCheck [12], and HLA3D [10] were discussed as conceptual predecessors, a direct head-to-head quantitative benchmark on the same allele set was not performed in this study. Methodological incompatibilities—specifically, that Snowflake utilizes neural-network SASA predictions without physicochemical charge/hydropathy filtering and lacks coverage for the HLA-DP locus [11], HLA-EpiCheck relies on computationally intensive molecular dynamics trajectories rather than static registry-defined eplets [12], and HLA3D functions primarily as a structural alignment and visualization toolkit [10]—precluded a unified comparative execution within our high-throughput pipeline. Future cross-platform benchmarking studies are warranted to systematically compare static AlphaFold 3 biophysical filtration against neural-network SASA predictors and dynamic simulation frameworks.

Ultimately, this pipeline represents an exploratory computational framework rather than a validated correction to existing clinical risk assessments. As an immediate next step, we call for multicenter retrospective validation studies utilizing existing high-resolution HLA-typed kidney transplant cohorts with documented longitudinal de novo DSA kinetics, biopsy-proven antibody-mediated rejection, and allograft survival data to empirically recalibrate structural thresholds. Both large-scale retrospective registry evaluations and subsequent outcome-based prospective clinical trials are strictly required before these structural profiles can be translated into donor selection protocols or individualized immunosuppression strategies.

## 4. Materials and Methods

### 4.1. Study Design and Population

An in silico computational study of simulated donor–recipient pairs was conducted to assess molecular compatibility. The virtual study cohort (*n* = 10,000 simulated diploid donor–recipient pairs) was generated via Monte Carlo random sampling with replacement using high-resolution HLA allele and haplotype frequencies for the European population from the Allele Frequency Net Database [35]. Because each simulated individual was assigned a diploid genotype (two alleles or haplotypes per locus) sampled proportionally to population frequencies, common genotypes recur across the 10,000 iterations to reflect real-world clinical encounter probabilities, whereas the effective sample size of unique biological donor–recipient diploid genotype combinations (*n*_unique_) within the cohort was 3277 for HLA-A, 4899 for HLA-B, 3727 for HLA-C, 2218 for HLA-DR, 2438 for HLA-DQ, and 4595 for HLA-DP. To ensure 100% structural coverage for downstream evaluation, the virtual cohort was strictly constrained to alleles present in the pre-computed AlphaFold structural library. Donor and recipient genotypes were simulated as independent random sampling events. Alleles for HLA-A, -B, -C, and -DRB1 were sampled independently within each locus based on their normalized European population frequencies (Supplementary Table S13), whereas for the heterodimeric class II loci (HLA-DQ and HLA-DP), the *α* (DQA1, DPA1) and *β* (DQB1, DPB1) chains were modeled jointly as functionally paired *α*/*β* haplotypes according to established European linkage disequilibria [31]. Because cross-locus linkage disequilibrium (e.g., extended ancestral haplotypes spanning HLA-A–B–C–DRB1) was not modeled during sampling, all downstream mismatch counts, biophysical sums, and reclassification proportions were evaluated strictly on an individual per-locus basis rather than summed into a pan-HLA total score, ensuring that within-locus marginal genotype distributions remain unaffected by cross-locus independence.

### 4.2. HLA Structural Modeling

High-precision 3D models of HLAs were generated using the AlphaFold 3 deep learning algorithm via the official AlphaFold Server web platform (https://alphafoldserver.com/, accessed on 20 September 2026) [15]. Modeling was performed for a curated reference panel of high-frequency alleles and functional *α*/*β* class II heterodimers (comprising 10 to 15 dominant allotypes or *α*/*β* haplotype combinations per locus with European population frequencies ≥ 1.0%; complete list and relative frequencies in Supplementary Table S13) selected from the Allele Frequency Net Database [35]. Together, this panel accounts for >85–92% of the cumulative allele and haplotype frequency distribution in the European demographic and captures the primary polymorphic eplet-defining positions across all six classical HLA loci. While this high-frequency panel provides a robust foundation for proof-of-concept population modeling, expanding the pre-computed structural library to hundreds of intermediate- and low-frequency alleles across diverse global ancestries would further enhance allelic granularity in future clinical implementations (as discussed in Section 3). For class II antigens, this selection ensures comprehensive coverage of the critical hypervariable regions, including the alpha-helical walls and peptide-binding groove pockets (e.g., DRB1 pockets 4, 7, and 9), which are primary targets for allorecognition.

Full-length amino acid sequences were retrieved from the IPD-IMGT/HLA database.

Structural predictions were executed via the AlphaFold 3 Server (Google DeepMind, Isomorphic Labs) using multimer modeling for both class I (heavy chain + *β*_2_-microglobulin) and class II (*α* chain + *β* chain) complexes in a standardized peptide-free state (without exogenous peptide sequences) to prevent sequence-specific peptide bias across the simulated cohort [23,33]. The standard modeling pipeline was utilized without custom templates, employing default settings for multiple sequence alignment depth. For each unique complex, the algorithm generated five independent model samples (seeds). Output models were evaluated based on the predicted pLDDT scores. The top-ranked structural prediction (Rank 1), corresponding to the highest combined confidence score, was selected for each allele, and AMBER relaxation was subsequently applied to all top-ranked models to minimize steric clashes of side chains.

Because the AlphaFold Server does not maintain permanent public run links, all generated .cif structural models (comprising the 76 simulated cohort alleles/haplotypes alongside 4 additional reference complexes in the 80-model repository; Supplementary Tables S6 and S13), confidence metadata, allele/haplotype frequency tables, and analysis scripts have been permanently deposited in Zenodo (version 2.1.0) (https://doi.org/10.5281/zenodo.23311701) and GitHub ((https://github.com/NefroStat/HLA-Structural-Immunogenicity), accessed on 20 October 2026) to ensure full computational reproducibility.

### 4.3. Structural Validation

To confirm the accuracy of AlphaFold 3 predictions, a comparative structural benchmarking against experimentally determined crystal structures from the PDB was conducted. Thirteen representative control structures (7 class I and 6 class II) with resolution ≤ 3.25 Å were selected [28]. Structural superimposition was performed using the PyMOL Molecular Graphics System (version 3.1.6.1) [36]. Accuracy was evaluated by calculating the RMSD for C*α* atoms between the predicted and experimental models.

### 4.4. Eplet Definition Algorithms

Eplet load for each donor–recipient pair was calculated using three comparative methodologies:

1. Standard method: eplet definition was based on linear comparison of amino acid sequences using the theoretical eplet repertoire according to the International HLA Epitope Registry database (version 3.0) [37], based on HLAMM algorithms [1,24]. All polymorphic residues were included.
2. Verified method: only antibody-verified eplets listed in the HLA epitope registry were included. This conservative approach excludes theoretically predicted but clinically unproven epitopes [38].
3. Dual-filter structural and biophysical method: a novel 3D structure-based filtration algorithm developed in this study. For each polymorphic residue identified as a potential mismatch, the SASA was calculated using the Shrake–Rupley algorithm [25] implemented in the Biopython library (version 1.81) [39]. To establish an empirical baseline for physicochemical reactivity, we derived biophysical reference thresholds from the subset of experimentally confirmed, antibody-verified eplets across the registry (*N* = 170 unique antibody-verified eplets out of 521 catalogued entries across the four registry sheets: *n* = 72 of 252 for class I [HLA-A, -B, -C] and *n* = 98 of 269 for class II [HLA-DR, -DQ, -DP]; Supplementary Table S9). Within this verified reference cohort (*N* = 170), the median [Q1; Q3] absolute net charge at pH 7.4 was 0.55 [0.44; 0.75] *e* and the median total hydropathy score was −2.35 [−4.30; 0.30]. We calculated the 5th-percentile values of this verified distribution—corresponding to an absolute charge cutoff of |*Q_net_*| ≥ 0.3326 e (0.3047 *e* for class I and 0.3326 *e* for class II; signed Qnet 5th percentile = −1.4408 *e*) and a total hydropathy cutoff of *H_total_* ≥ −7.265 (−8.070 for class I and −5.385 for class II; |*H_total_*| 5th percentile = 0.400)—to evaluate the relative contribution of spatial accessibility versus physicochemical gating in a dedicated ablation analysis (Supplementary Table S9).

Selection criteria: A predicted mismatch was retained in the final functional eplet load only if it successfully passed two sequential constraints: (1) spatial accessibility, and (2) biophysical reactivity. Because SASA was calculated at the individual residue level, the baseline steric accessibility of an eplet was determined by evaluating the primary polymorphic anchor position defining its registry nomenclature (e.g., position 62 for eplet 62QE). If this primary defining residue met the SASA ≥ 10.0 Å^2^ threshold—which represents the minimum solvent-exposed surface area required for a side chain to accommodate at least one water molecule (probe radius 1.4 Å) or form a direct contact with an antibody CDR loop—the eplet was classified as sterically accessible [20,21,22]. To verify that this single-anchor rule and the 10.0 Å^2^ cutoff did not introduce methodological artifacts for multi-residue eplets, we conducted two dedicated sensitivity analyses across all 10,000 simulated pairs (Supplementary Tables S10 and S11): (1) varying the SASA threshold across 5.0, 10.0, 15.0, and 20.0 Å^2^, and (2) comparing the primary-residue rule against an inclusive “Any-residue (OR)” rule (retaining a multi-residue eplet if at least one constituent residue exceeds 10.0 Å^2^) and a restrictive “All-residues (AND)” rule (requiring every constituent residue in the patch to exceed 10.0 Å^2^). Both sensitivity analyses confirmed that the primary-residue rule at 10.0 Å^2^ closely matches the inclusive “Any-residue (OR)” patch definition across all loci while preserving the class I versus class II reclassification dichotomy across the entire 5.0–20.0 Å^2^ threshold spectrum (Supplementary Tables S10 and S11). Residues failing either the steric or the biophysical constraint were classified as “cryptic” or “biophysically inert” and excluded from the final load calculation [26,27].

### 4.5. Physicochemical Profiling

For the polymorphic residues defining each identified eplet mismatch, two key biophysical parameters affecting theoretical immunogenicity were calculated:

Electrostatic charge:

Unlike simplified integer charge models, the net electric charge of the eplet was calculated at physiological pH 7.4 using the Henderson–Hasselbalch equation [40]. This method accounts for partial ionization of amino acid side chains and terminal groups.

The net charge (*Q_net_*) was defined as the sum of partial charges of positively and negatively ionized groups according to the formula:

$$Q_{\mathrm{net}}=\;\sum_{i}^{N_{\mathrm{pos}}}\left(\frac{1}{1+10^{\mathrm{pH}-pK_{a,i}}}\right)-\;\sum_{j}^{N_{\mathrm{neg}}}\left(\frac{1}{1+10^{pK_{a,j}-\mathrm{pH}}}\right)$$

where p*K_a_* represents the standard unperturbed acid dissociation constants of the ionizable functional groups of amino acid residues in aqueous solution (Asp: 3.86, Glu: 4.25, His: 6.00, Tyr: 10.07, Lys: 10.53, Arg: 12.48, Cys: 8.33; N-terminus: 9.69, C-terminus: 2.34) as defined in the Bio.SeqUtils.ProtParam (ProteinAnalysis.charge_at_pH) and Bio.SeqUtils.IsoelectricPoint (positive_pKs and negative_pKs dictionaries) modules of Biopython (version 1.81) [39,40]. Within this high-throughput pipeline, ionization states were computed using these intrinsic reference p*K_a_* constants without modeling local protein environment-induced shifts in protolytic equilibria [17,18,39].

For the final load assessment, the sum of absolute charge values of all mismatched eplets was used ($Q_{\mathrm{total}}=\sum \left|Q_{\mathrm{net}}\right|$). Because metrics were calculated per individual eplet and subsequently summed, the physicochemical contributions of polymorphic residues shared across multiple overlapping eplets were repeatedly included in the cumulative scores. This aligns with the underlying HLAMM premise that each eplet acts as an independent theoretical antibody recognition target.

Total hydrophobicity potential:

The hydrophobic profile was calculated based on the Kyte–Doolittle hydropathy scale [18,30] using the ProteinAnalysis.gravy method in Bio.SeqUtils.ProtParam (Biopython version 1.81) [39]. Since eplets vary in length, using the simple sequence-averaged index—the grand average of hydropathicity—may obscure the cumulative contribution of extensive hydrophobic patches [30]. Therefore, the total hydrophobic potential (*H_total_*) was calculated to reflect the total hydrophobic surface area available for interaction:

$$H_{\mathrm{total}}=\;\sum_{i=1}^{L}h_{i}$$

where *h_i_* is the hydrophobicity index of the *i*-th amino acid residue and *L* is the length of the eplet sequence. This approach provides a length-weighted heuristic index of cumulative patch hydropathy that accounts for motif size, without modeling continuum solvation free energies.

### 4.6. Fixed-Cutoff Load Categorization

To assess the clinical significance of changes in eplet load, donor–recipient pairs were classified into computational load categories according to thresholds validated in previous clinical studies [2,3]:

Match: 0 eplet mismatches.

Intermediate/Low Risk: 1–9 eplet mismatches.

High Risk: ≥10 eplet mismatches per molecule.

This scale was used to calculate the threshold-crossing proportion when transitioning from the standard method to the structural approach.

### 4.7. Statistical Analysis

Statistical analyses were performed using the R computing environment (version 4.5.1) [41]. Because eplet mismatch counts and cumulative physicochemical scores represent discrete, bounded, and inherently skewed distributions (also confirmed via the Anderson–Darling test in the ‘nortest’ package (version 1.0-4) [42]), non-parametric descriptive statistics—median and interquartile range (Median [Q1; Q3])—were selected a priori as the appropriate summary metrics. Because the dual-filter structural method and the verified-only method represent sequential subset filtrations of the baseline HLAMM repertoire, the directional reduction in mismatch counts and cumulative charge is an inherent deterministic property of the algorithm. Therefore, formal null-hypothesis significance testing (*p*-values) was not applied to comparisons between the three eplet definitions. Instead, to descriptively quantify the magnitude of the algorithmic shifts across paired observations, we calculated Hodges–Lehmann paired median differences with 95% confidence intervals [43] and rank-biserial correlation effect sizes (*r*) based on paired Wilcoxon signed-rank statistics [44]. The strength and direction of the associations between eplet mismatch counts, cumulative electrostatic charge, and cumulative hydropathy were assessed using Spearman’s rank correlation coefficient (ρ) rather than Pearson’s linear correlation coefficient. Spearman’s rank-based approach was selected a priori because the evaluated metrics violate bivariate normality due to discrete integer scaling (for eplet counts), lower-bound censoring at zero for matched pairs (floor effects in |*Q_net_*| and count), skewed distributions with heavy tails (as evidenced by the outlier profiles in Figure 2 and Figure 4 and the Anderson–Darling test, *p* < 0.05), and non-linear monotonic scaling across loci. Categorical agreement across fixed numerical cutoffs between the standard 1D method and our dual-filter structural model was quantified using Cohen’s quadratic weighted kappa coefficient (*κ_w_*) with two-sided 95% confidence intervals [45]. All statistical tests were two-tailed, and significance was defined as *p* < 0.05. Data visualization was generated using the ‘ggplot2’ package (version 3.5.1) [46]. Because the 10,000 simulated pairs were generated via frequency-weighted sampling with replacement from a finite allele library, confidence intervals and correlation *p*-values computed on the full cohort (*n* = 10,000) reflect population-weighted precision rather than strictly independent observations. To verify that genotype duplication and resampling from a finite structural library did not artificially inflate statistical significance or distort uncertainty estimates, we conducted two complementary sensitivity analyses: (1) restricting all estimates strictly to deduplicated, unique biological donor–recipient diploid genotype combinations per locus (*n_unique_* ranging from 2218 to 4899; Supplementary Table S7), and (2) performing a non-parametric allele-level cluster bootstrap (resampling the structural allele/haplotype clusters with replacement across 1000 iterations) to treat the structural allele library itself as the primary unit of uncertainty (Supplementary Table S5).

## 5. Conclusions

Incorporation of AlphaFold 3 into histocompatibility assessment enables a transition from linear, sequence-based matching to high-resolution structural profiling. By integrating biophysical constraints—solvent accessibility, electrostatic potential, and hydrophobicity—our pipeline algorithmically filters theoretically identified eplets, particularly for the complex HLA class II loci. The practical implementation of this approach is no longer limited by computational barriers. The generation and storage of pre-computed structural libraries allow for the rapid computational assessment of donor–recipient pairs without requiring high-performance computing at the point of care. By making these algorithms and structural databases accessible, we support the development of structural histocompatibility profiling. Ultimately, we hypothesize that this computational framework provides a more comprehensive biophysical characterization of HLA mismatches. However, rigorous retrospective validation across longitudinal clinical transplant cohorts, followed by prospective clinical trials, is strictly necessary to determine whether these theoretically refined structural profiles correlate with actual alloimmune and graft survival outcomes.

## Figures and Tables

**Table 1:** Benchmarking AlphaFold 3 predictive accuracy against experimental PDB structures.

HLA Locus	Allele Name	PDB ID (Reference)	Resolution (Å)	AF3 pLDDT	AF3 ipTM/pTM	RMSD (Cα) [Å]
Class I	HLA-A*02:01	1DUZ	1.80	85.98	0.94/0.81	0.64
	HLA-A*24:02	3I6L	2.40	85.21	0.94/0.81	0.75
	HLA-B*07:02	6ULI	1.88	86.69	0.93/0.81	0.83
	HLA-B*27:05	1HSA	2.10	86.85	0.94/0.82	0.72
	HLA-B*51:01	1E27	2.20	87.41	0.94/0.82	0.29
	HLA-C*04:01	1QQD	2.70	85.50	0.94/0.81	0.87
	HLA-C*06:02	5W6A	2.90	86.25	0.94/0.80	0.67
Class II	HLA-DRB1*01:01	1DLH	2.80	87.28	0.79/0.79	0.36
	HLA-DRB1*04:01	5NI9	2.15	87.53	0.80/0.80	0.38
	HLA-DRB1*15:01	1BX2	2.60	87.34	0.79/0.79	0.38
	HLA-DQA1*05/DQB1*02	1S9V	2.22	85.67	0.79/0.79	0.36
	HLA-DQA1*03/DQB1*03	1JK8	2.40	86.30	0.80/0.79	0.40
	HLA-DPA1*01/DPB1*04	3LQZ	3.25	85.79	0.76/0.77	0.59

Comparison of predicted models against high-resolution PDB reference structures. Individual Cα RMSD values are rounded to two decimal places from the raw PyMOL alignment outputs shown in Figure 1 and Supplementary Figure S2. Across the 13 benchmarked structures (class I: 0.68 ± 0.19 Å, *n* = 7; class II: 0.41 ± 0.09 Å, n = 6; overall mean: 0.56 ± 0.20 Å), full-length AlphaFold 3 pLDDT scores inversely correlated with Cα RMSD (Spearman’s ρ = −0.560, *p* = 0.0463).

**Table 2:** Comparative analysis of eplet mismatch loads across HLA loci.

HLA Locus (n = 10,000)	Standard (HLAMM)	Structural (AlphaFold 3)	Verified Only
HLA-A	19 [12; 26]	17 [10; 23]	6 [3; 8]
HLA-B	22 [15; 29]	18 [12; 24]	5 [3; 7]
HLA-C	12 [7; 17]	10 [7; 15]	3 [1; 5]
HLA-DR	15 [10; 19]	11 [7; 14]	6 [3; 8]
HLA-DQ	12 [6; 17]	9 [4; 14]	6 [3; 10]
HLA-DP	7 [3; 12]	6 [2; 9]	2 [0; 3]

Note: Values are presented as median and interquartile range (Median [Q1; Q3]) for *n* = 10,000 simulated donor–recipient pairs evaluated per locus. Because the Structural (AlphaFold 3) and Verified Only repertoires are filtered subsets of the Standard (HLAMM) repertoire, the reduction in mismatch count is a deterministic property of the algorithm.

**Table 3:** Physicochemical profiling of mismatch loads across HLA loci.

**Electrostatic Charge**					
Locus	Standard (HLAMM)	Structural (AlphaFold 3)	Verified only	Δ Median (95% CI) [HLAMM vs. AF]	Effect Size (*r*)
HLA-A	13.69 [9.07; 18.44]	12.62 [8.03; 16.86]	4.74 [2.83; 6.66]	1.39 [1.36; 1.42]	0.847
HLA-B	16.37 [10.91; 22.18]	13.10 [8.23; 17.93]	3.33 [1.88; 4.77]	3.21 [3.17; 3.25]	0.863
HLA-C	7.05 [4.21; 9.87]	6.03 [3.58; 8.43]	1.98 [0.83; 3.00]	1.32 [1.32; 1.33]	0.807
HLA-DR	9.17 [5.81; 12.52]	7.28 [4.32; 9.98]	4.21 [2.22; 6.32]	2.01 [1.98; 2.03]	0.861
HLA-DQ	6.53 [3.58; 9.53]	5.14 [2.36; 7.67]	3.19 [1.33; 5.52]	1.60 [1.57; 1.63]	0.845
HLA-DP	5.69 [2.62; 9.18]	4.85 [1.83; 7.74]	1.33 [0.00; 3.95]	1.24 [1.18; 1.24]	0.842
**Hydrophobicity Potential**					
HLA-A	−39.70 [−56.02; −20.10]	−41.40 [−59.00; −21.70]	−22.90 [−31.50; −9.10]	2.75 [2.65; 2.80]	0.472
HLA-B	−30.70 [−47.30; −14.40]	−26.10 [−39.40; −11.90]	−5.00 [−12.30; 0.00]	−5.10 [−5.30; −4.90]	0.516
HLA-C	−11.90 [−21.40; −2.50]	−10.50 [−20.40; −3.50]	−5.60 [−11.20; 0.00]	−0.55 [−0.75; −0.45]	0.122
HLA-DR	−15.20 [−27.50; −2.00]	−16.30 [−26.50; −4.78]	−6.90 [−12.90; −1.70]	0.55 [0.40; 0.70]	0.069
HLA-DQ	−5.90 [−16.80; 0.00]	−5.50 [−16.60; 0.50]	−5.70 [−12.40; 0.00]	−1.90 [−2.05; −1.70]	0.180
HLA-DP	1.90 [−1.80; 6.50]	−0.20 [−5.90; 1.90]	−3.50 [−7.10; 0.00]	4.70 [4.65; 4.85]	0.689

Note: Values are presented as median [Q1; Q3] (*n* = 10,000 paired observations per locus). To quantify the magnitude of the deterministic algorithmic shift between the Standard (HLAMM) and Structural (AlphaFold 3) definitions, 95% confidence intervals for the paired median differences (Hodges–Lehmann estimates) and standardized effect sizes (rank-biserial correlation, r) are reported.

## Data Availability

The structural and physicochemical datasets generated in this study are provided as Source Data within this paper. Publicly available datasets used in this study include the IPD-IMGT/HLA Database (https://www.ebi.ac.uk/ipd/imgt/hla/ accessed on 20 October 2026), the EpRegistry database (https://www.epregistry.com.br/ accessed on 20 October 2026) for verified and potential eplet configurations, and the RCSB Protein Data Bank (https://www.rcsb.org/) for structural benchmarking. The full library of AlphaFold 3 structural models generated in this study is publicly available in the Zenodo repository (https://doi.org/10.5281/zenodo.23311701). The complete code used for structural modeling, physicochemical profiling, and statistical analysis is available in the GitHub repository ((https://github.com/NefroStat/HLA-Structural-Immunogenicity), accessed on 20 October 2026) and is permanently archived with the identifier DOI: https://doi.org/10.5281/zenodo.23311701. Transplant centers and research groups interested in applying this computational pipeline to retrospective clinical cohorts are encouraged to contact the corresponding author (V.B.) for collaborative research and technical integration.

## Supplementary Materials

The following supporting information can be downloaded at https://www.mdpi.com/article/10.3390/ijms27219442/s1.
