# Supplementary material for: Structural and Biophysical Profiling of HLA Class II Mismatches Using AlphaFold 3: A Computational Study
Source: Int J Mol Sci. 2026 Oct 23;27(21):9442. doi: 10.3390/ijms27219442 (PMC13763352; doi:10.3390/ijms27219442)
Supplement: Supplementary file 1 [file ijms-27-09442-s001.zip › Supplementary_Information.pdf]

Supplementary Information

This file and the accompanying Supplementary Data spreadsheet (SupplementaryData.xlsx) include:

- **Supplementary Figure S1:** Relationship between eplet mismatch count and cumulative heuristic physicochemical indices across HLA loci.
- **Supplementary Figure S2:** Complete 13-allele structural benchmarking overlay of AlphaFold 3 models against experimental PDB reference structures.
- **Supplementary Table S1 (Table S1 Desc Stats in Excel):** Descriptive statistics (Median, Q1, Q3, IQR) and paired Hodges–Lehmann median differences with rank-biserial effect sizes (r) across all HLA loci, methods, and metrics (n = 10,000).
- **Supplementary Table S2 (Table S2 Correlations in Excel):** Spearman's rank correlations between cumulative electrostatic charge and hydropathy across HLA loci.
- **Supplementary Table S3 (Table S3 Fig S1 Corr in Excel):** Spearman's rank correlations between structural eplet count and cumulative physicochemical indices.
- **Supplementary Table S4 (Table S4 Reclassification in Excel):** Computational load reclassification summary and Wilson 95% confidence intervals for baseline high-load pairs (≥10 eplets).
- **Supplementary Table S5 (shown below and in Excel):** Categorical agreement (quadratic weighted Cohen's kappa, κw) with corrected two-sided 95% confidence intervals across the full cohort (n = 10,000), deduplicated unique diploid genotype combinations (nunique), and allele-level cluster bootstrap (B = 1,000).
- **Supplementary Table S6 (Allele Library & Model Quality in Excel):** Internal AlphaFold 3 confidence metrics (pLDDT, ipTM, pTM, Ranking Score) across the modeled structural library and per-locus summary statistics.
- **Supplementary Table S7 (Table S7 Unique Genotypes in Excel):** Sensitivity analysis restricted strictly to deduplicated, unique biological donor–recipient diploid genotype combinations per locus (nunique = 2,218 to 4,899).
- **Supplementary Table S8 (Table S8 Range Matched Corr in Excel):** Range-matched 1:1 count-distribution subsampling (n = 2,861 per locus across counts 1–18) and partial Spearman rank correlations conditioned on eplet count.
- **Supplementary Table S9 (Table S9 Ablation Study in Excel):** Five-arm component ablation study isolating spatial accessibility (SASA ≥ 10.0 Å²) versus the 5th-percentile verified biophysical gate (|Qnet| ≥ 0.3326 e and Htotal ≥ -7.265; N = 170 verified eplets).
- **Supplementary Tables S10 & S11 (Table S10 SASA Sensitivity & Table S11 Patch Rules in Excel):** SASA threshold sensitivity analysis (5.0, 10.0, 15.0, and 20.0 Å²) and multi-residue patch rule comparison (Primary-anchor vs. Any-residue [OR] vs. All-residues [AND]).

- **Supplementary Table S12 (Table S12 Histidine pKa in Excel):** Sensitivity analysis of histidine ionization microenvironments (pKa 5.5–7.0) and signed versus absolute biophysical thresholds.
- **Supplementary Table S13 (Table S13 Allele Frequencies in Excel):** Complete list of simulated European HLA alleles and α/β heterodimeric haplotypes with population and empirical sample frequencies

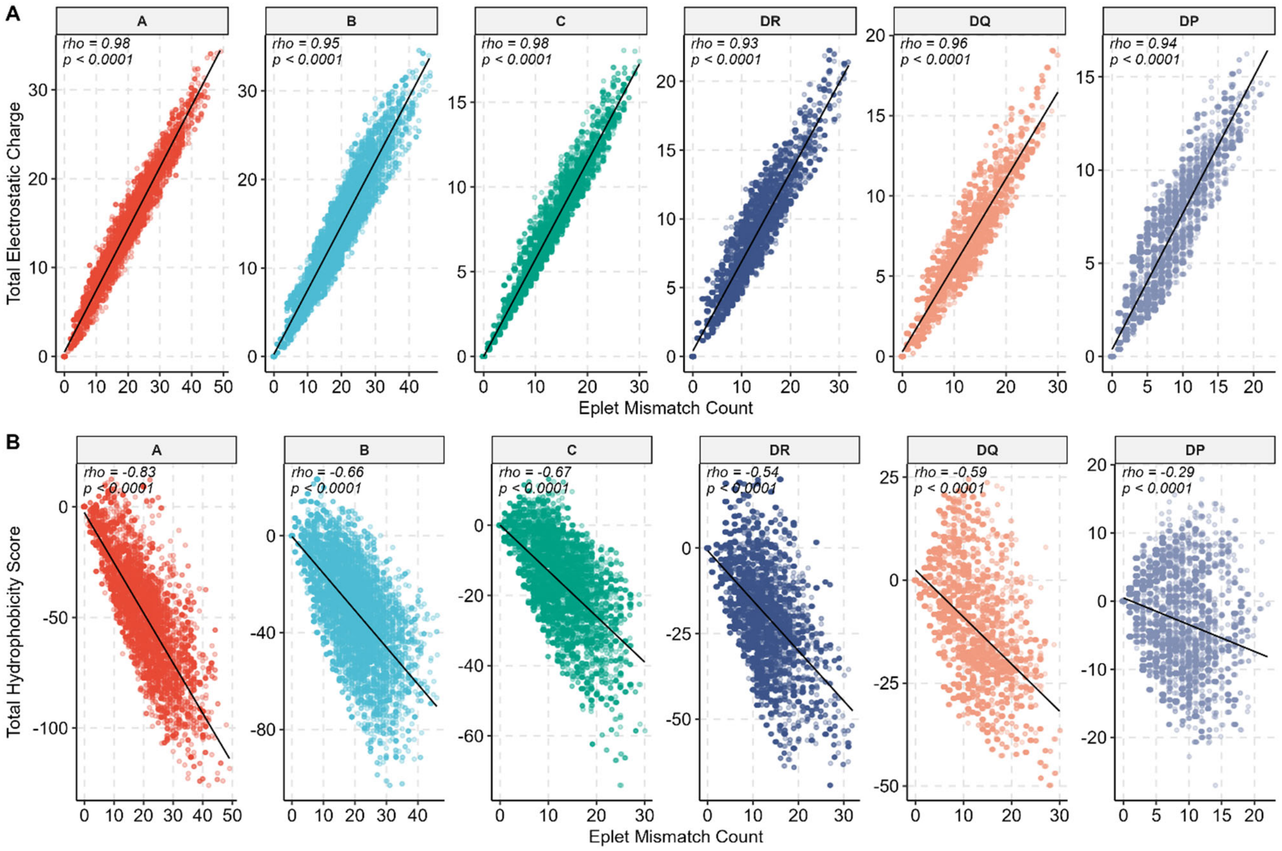

Supplementary Figure S1. Relationship between eplet mismatch count and cumulative heuristic physicochemical indices across HLA loci.

Scatter plots illustrate the relationship between the number of structural mismatched eplets (X-axis) and cumulative heuristic physicochemical indices (Y-axis) across n = 10,000 simulated donor–recipient pairs per locus.

(A) Eplet Count vs. Cumulative Absolute Charge ($Qtotal = \sum |Qnet|$). *Because cumulative charge sums non-negative absolute point charges across all mismatched eplets (including shared residues in overlapping eplets), a strong positive correlation is observed algebraically across all loci (Spearman's ρ = 0.926–0.978, p < 0.0001).*

(B) Eplet Count vs. Cumulative Hydropathy Score (Htotal). *Because Kyte–Doolittle hydropathy is a signed quantity summing both hydrophilic (negative) and hydrophobic (positive) substitutions, HLA class I loci exhibit strong inverse correlations (ρ = -0.579 to -0.832, p < 0.0001), whereas the HLA-DP locus exhibits only a weak association both in the full cohort (ρ = -0.292, p < 0.0001) and in the 1:1 count-matched subsample (ρmatched = -*

0.138, $p = 1.33 \times 10^{-13}$; Supplementary Table S8), indicating that signed patch hydropathy varies largely independently of total eplet count at HLA-DP.

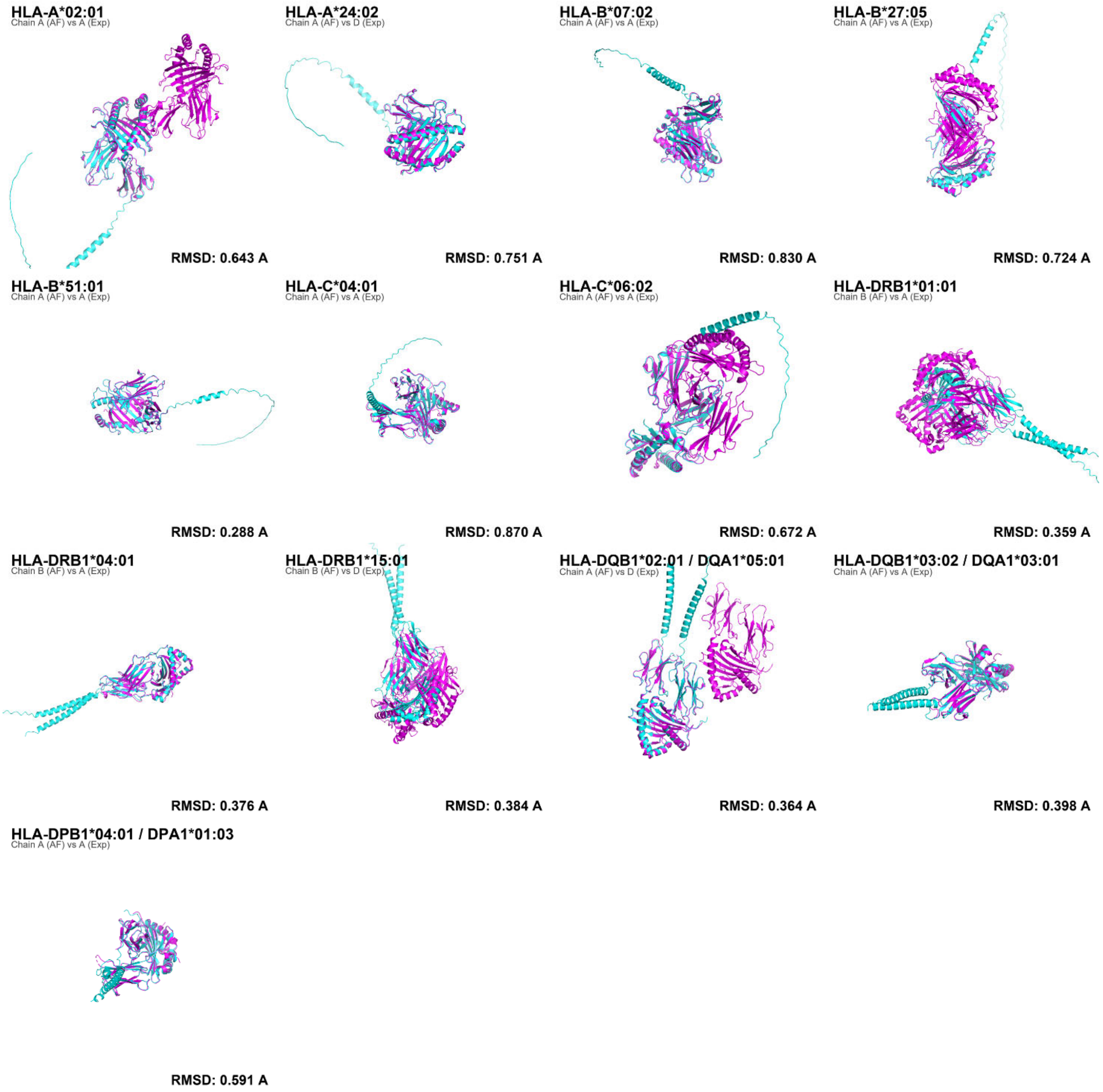

Supplementary Figure S2. Complete 13-allele structural benchmarking of AlphaFold 3 models against experimental PDB crystal structures.

Superimposition of all 13 benchmarked AlphaFold 3 models (cyan; 7 class I and 6 class II complexes) onto their corresponding high-resolution X-ray crystallographic reference structures from the Protein Data Bank (purple). Unrounded Cα RMSD values (in Å) generated directly by PyMOL across the aligned extracellular domains are displayed on each panel (rounded to two decimal places alongside AlphaFold 3 confidence metrics in Table 1 of the main text).

Supplementary Table S5 (formerly Supplementary Table S1). Categorical agreement between Standard (HLAMM) and dual-filter Structural (AlphaFold 3) computational load classifications across full, deduplicated, and allele-bootstrapped cohorts.

HLA Locus	Full Cohort Kw (n = 10,000)	Full Cohort Two-Sided 95% CI	Unique Diploid Pairs (nunique)	Deduplicated Kw, unique	Deduplicated Two-Sided 95% CI
**HLA-A**	0.948	0.943–0.954	3,277	0.884	0.864–0.905
**HLA-B**	0.835	0.821–0.849	4,899	0.831	0.809–0.853
**HLA-C**	0.879	0.870–0.887	3,727	0.851	0.835–0.868
**HLA-DR**	0.715	0.701–0.729	2,218	0.731	0.700–0.761
**HLA-DQ**	0.857	0.849–0.865	2,438	0.824	0.804–0.844
**HLA-DP**	0.789	0.778–0.799	4,595	0.748	0.731–0.765

Note: *Categorical agreement across fixed numerical cutoffs (0 eplets [Match], 1–9 eplets [Intermediate/Low Load], and ≥10 eplets [High Load]) was quantified using quadratic weighted Cohen's kappa coefficient (κw) with analytical two-sided 95% confidence intervals. To account for non-independence arising from Monte Carlo resampling of the finite allele library, κw was additionally evaluated on strictly deduplicated unique biological donor–recipient diploid genotype combinations per locus (nunique) and via a non-parametric allele-level cluster bootstrap (resampling donor allele/haplotype clusters with replacement across B = 1,000 iterations). Interpretation of agreement strength: <0.20 Slight, 0.21–0.40 Fair, 0.41–0.60 Moderate, 0.61–0.80 Substantial, 0.81–1.00 Almost Perfect.*
